# Performance Study of Compact Semiconductor Neutron Spectrometer HardPix for Lunar Water Mapping

**DOI:** 10.3390/s26165256

**Published:** 2026-08-19

**Authors:** Robert Filgas, Daniel Matthiä, Hugo Cintas, Tomáš Slavíček, Jindřich Jelínek, Stefan Gohl, Milan Malich, Hugo Natal da Luz, Benedikt Bergmann, Thomas Berger, Francesca McDonald, Giovanni Santin

**Affiliations:** 1 Institute of Experimental and Applied Physics, Czech Technical University in Prague, 110 00 Prague, Czech Republic; hugo.cintas@cvut.cz (H.C.); tomas.slavicek@cvut.cz (T.S.); jindrich.jelinek@cvut.cz (J.J.); stefan.gohl@cvut.cz (S.G.); milan.malich@cvut.cz (M.M.); hugo.nluz@cvut.cz (H.N.d.L.); benedikt.bergmann@cvut.cz (B.B.); 2 German Aerospace Center (DLR), Institute of Aerospace Medicine, 51147 Cologne, Germany; daniel.matthiae@dlr.de (D.M.); thomas.berger@dlr.de (T.B.); 3 Department of Nuclear and Particle Physics, University of Geneva, CH-1211 Geneva, Switzerland; 4 European Space Agency, Keplerlaan 1, 2201 AZ Noordwijk, The Netherlands; francesca.mcdonald@esa.int (F.M.); giovanni.santin@esa.int (G.S.)

**Keywords:** neutron spectrometer, Timepix, HardPix, hydrogen, lunar water ice, cosmic radiation, albedo neutrons

## Abstract

**Highlights:**

**What are the main findings?**

- As estimated by the model, neutron fluence rates on the lunar surface strongly correlate with water content, with epithermal neutron flux decreasing by up to ~85% and total flux by ~60% at 10% water content.
- Neutron HardPix achieves practical detection capability, enabling identification of ~2–3% water variations in <2 h under realistic lunar conditions.

**What are the implications of the main findings?**

- Miniaturized semiconductor neutron spectrometers can replace traditional ^3^He-based systems on small commercial space missions.
- Neutron HardPix enables localized resource prospecting essential for Artemis-era surface operations.

**Abstract:**

The current interest in lunar exploration led by the Artemis program is pushing scientists to search for lunar water deposits directly on the surface of the Moon, using small robotic rovers. The Institute of Experimental and Applied Physics, Czech Technical University, Prague (IEAP CTU), is developing a miniature Timepix3-based detector called Neutron HardPix, which is capable of mapping water deposits using non-invasive detection of neutrons created underground by cosmic rays and thermalized by hydrogen. This neutron spectrometer measures count rate variations in thermal, epithermal and fast neutrons attributed to hydrogen abundance in the lunar subsurface, while monitoring cosmic radiation as a natural source of neutrons. Neutron HardPix is based on the miniature (<0.1 U, 130 g) radiation monitor HardPix, and has significant space heritage.

## 1. Introduction

**Figure 1:**
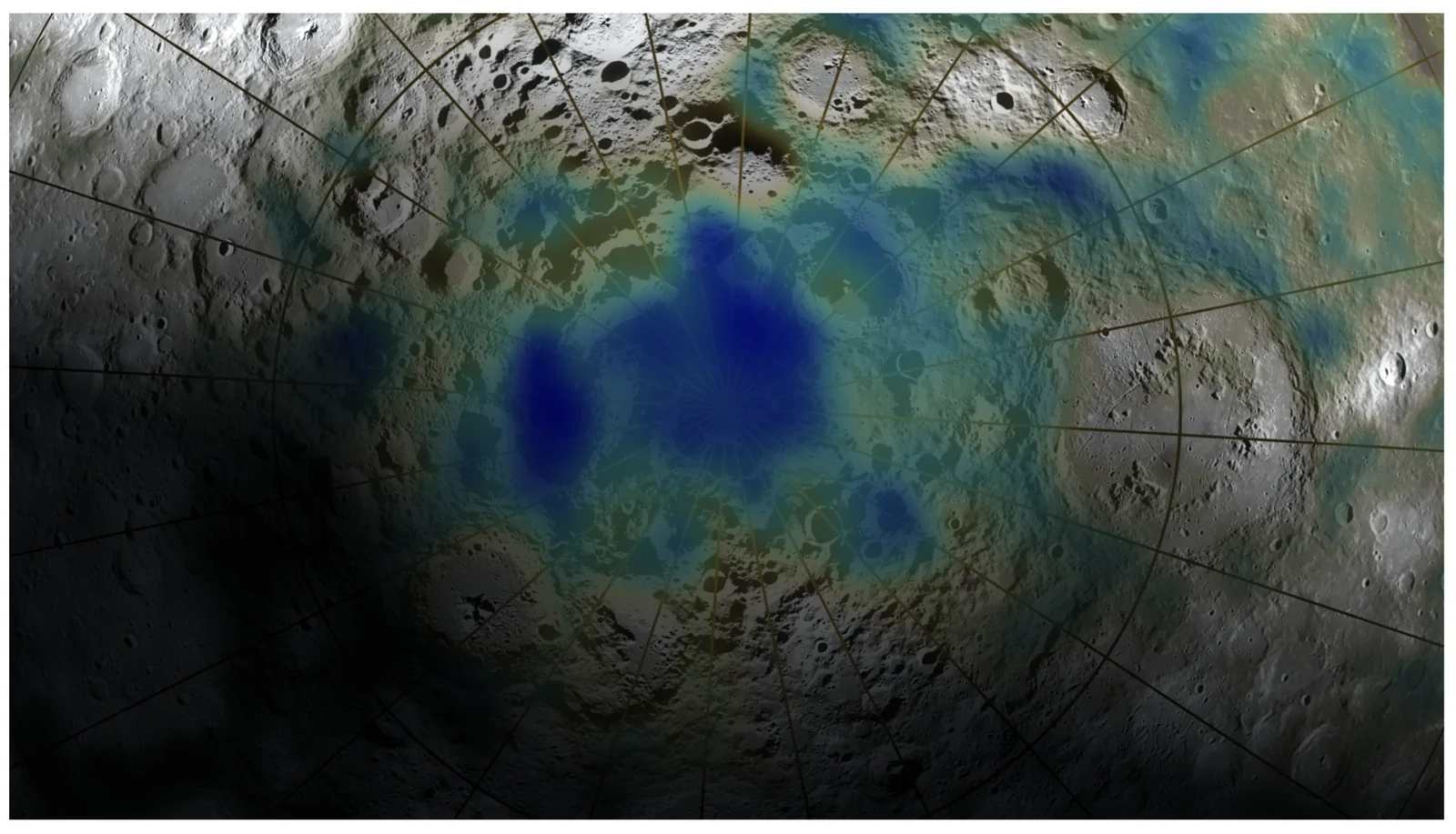
Hydrogen concentration near the lunar south pole derived from Lunar Prospector neutron measurements. Blue regions indicate enhanced hydrogen abundance consistent with possible hydrogen-bearing volatiles and water ice deposits. Image credit: NASA Goddard Space Flight Center Scientific Visualization Studio (SVS ID 3480).

The hypothesis that water may exist as ice at the lunar poles was first advanced several decades ago [1,2]. Permanently shadowed regions (PSRs) near the lunar poles are characterized by extremely low temperatures, which are estimated to have remained below approximately 100 K for much of the Moon’s geological history, thereby providing conditions favorable for the long-term stability of water ice [3,4]. Subsequent lunar orbital investigations have provided further indirect evidence supporting this hypothesis. Measurements from bistatic radar experiment aboard the National Aeronautics and Space Administration’s (NASA, www.nasa.gov) Clementine mission and from a neutron spectrometer on board the Lunar Prospector (Figure 1) have indicated enhanced hydrogen concentrations in the polar regions, consistent with the presence of hydrogen-bearing volatiles such as water ice [5,6]. More recent observations from the Lunar Exploration Neutron Detector onboard NASA’s Lunar Reconnaissance Orbiter have reinforced the interpretation that significant quantities of water ice may be present within lunar PSRs [7,8,9].

In addition, orbital imaging and analyses of lunar samples suggest the presence of hydrogen-rich material in localized areas near the poles, potentially associated with water ice deposits. However, the spatial resolution of existing remote sensing remains insufficient to identify the physical state, distribution, and concentration of these hydrogen-bearing materials. As a consequence, current knowledge of lunar water resources does not yet support reliable assessment or planning for resource extraction. These limitations underscore the necessity of future exploratory missions capable of conducting in situ measurements at a local scale in order to perform detailed hydrogen mapping and directly characterize potential subsurface and surface volatile deposits.

### 1.1. Hydrogen Detection Using Neutrons

Neutron-based techniques have long been employed for the determination of moisture content in large volumes of geological materials. These methods exploit the strong interaction between neutrons and hydrogen, which is abundant in water molecules. Hydrogen is particularly effective at moderating fast neutrons through elastic scattering, thereby converting them into epithermal and ultimately thermal neutrons. Measurements of the resulting thermalized neutron flux therefore provide the fundamental observational basis for estimating the water or hydrogen content within a target material.

On the Moon, high-energy neutrons are continuously generated in the subsurface as a result of interactions between galactic cosmic rays (GCRs) and regolith nuclei. Once produced, these neutrons propagate through the lunar regolith, where they undergo multiple scattering events and may be removed from the neutron population through capture reactions. The presence of hydrogen in the subsurface significantly enhances neutron moderation efficiency.

### 1.2. Neutron Spectrometers

To date, the majority of flight-qualified neutron spectrometers have relied on ^3^He gas-filled proportional counters. Representative examples include the Dynamic Albedo of Neutrons (DAN) instrument onboard the Curiosity rover [10], the Neutron Spectrometer on the Lunar Prospector [11] (Figure 1), the High Energy Neutron Detector (HEND) aboard Mars Odyssey [12], the Fine Resolution Epithermal Neutron Detector (FREND) onboard ESA’s Trace Gas Orbiter (TGO) [13], the Mercury Gamma-ray and Neutron Spectrometer (MGNS) on BepiColombo [14], and the Lunar Exploration Neutron Detector (LEND) aboard the Lunar Reconnaissance Orbiter [15].

Despite their proven scientific performance and extensive flight heritage, a significant proportion of the existing instruments has been developed by the Space Research Institute of the Russian Academy of Sciences (IKI), while many of the remaining non-Russian instruments originate from U.S. national laboratories, making them subject to export control regulations such as the International Traffic in Arms Regulations (ITAR). These factors complicate access to such technologies for international and commercial space missions. In addition, the integration of ^3^He gas proportional counters onto small spacecraft platforms presents non-negligible engineering challenges. These detectors use pressurized gas and require high-voltage biasing for charge collection, both of which introduce potential risks in terms of mechanical safety, system reliability, and mission qualification.

In recent years, semiconductor detectors have gained increasing popularity in space applications owing to their compact size, low power consumption, and reduced cost. When coupled with an appropriate neutron conversion layer, such detectors can function effectively as neutron spectrometers. An example of this approach is the Lunar Lander Neutron and Dosimetry (LND) experiment aboard the Chang’E-4 lander [16].

### 1.3. HardPix Neutron Spectrometer

The Institute of Experimental and Applied Physics, Czech Technical University, Prague (IEAP CTU, www.utef.cvut.cz), has extensive experience in the development of silicon detectors optimized for the detection of both thermal and fast neutrons. The use of technologically advanced pixel detectors of the Timepix family, adapted for neutron detection through the application of suitable neutron conversion layers, has been proposed and systematically investigated in a number of publications. References [17,18] provided comprehensive information on these developments, including their deployment within the Toroidal LHC ApparatuS (ATLAS) experiment at the Large Hadron Collider (LHC) at the European Organization for Nuclear Research (CERN, home.cern). These studies also reported on extensive experimental campaigns conducted at the Los Alamos Neutron Science Center (LANSCE), where detailed performance tests of the detectors were carried out under well-characterized neutron fields.

IEAP CTU, in collaboration with partner institutions, has also developed a number of radiation-tracking instruments based on Timepix chips that have been successfully deployed in space applications. Timepix is a hybrid active pixel detector developed within the Medipix Collaboration at CERN. Its core component is a semiconductor sensor layer segmented into a matrix of 256 × 256 pixels with a pixel pitch of 55 µm, corresponding to an active area of approximately 1.4 × 1.4 cm^2^. This sensor layer is bump-bonded to the readout’s application-specific integrated circuit (ASIC). The detector operates without the need for active cooling, relying solely on passive heat dissipation.

Timepix detectors can record detailed tracks of ionizing particles, corresponding to their trajectories within the solid-state pixel sensor. Track shapes can differ between particle types, energy, and angle, allowing discrimination of particle species, determination of incident direction, and measurement of ionization energy loss in the detector. Particle identification is performed using a combination of track morphology and stopping-power information, enabling effective separation of electrons, protons, and heavier ions—particle species that are of primary relevance in the space radiation environment [19].

Since 2012, several IEAP CTU miniature radiation monitors have been deployed aboard the International Space Station (ISS) in collaboration with the University of Houston and NASA [20,21,22]. A key milestone in demonstrating the suitability of Timepix technology for space applications was the Space Application of Timepix-based Radiation Monitor (SATRAM, satram.utef.cvut.cz) project [23,24]. SATRAM operates as a remotely controlled spacecraft radiation monitor aboard the European Space Agency (ESA, www.esa.int) Proba-V satellite, which has been in a near-polar Earth orbit at an altitude of approximately 820 km since its launch in May 2013.

**Figure 2:**
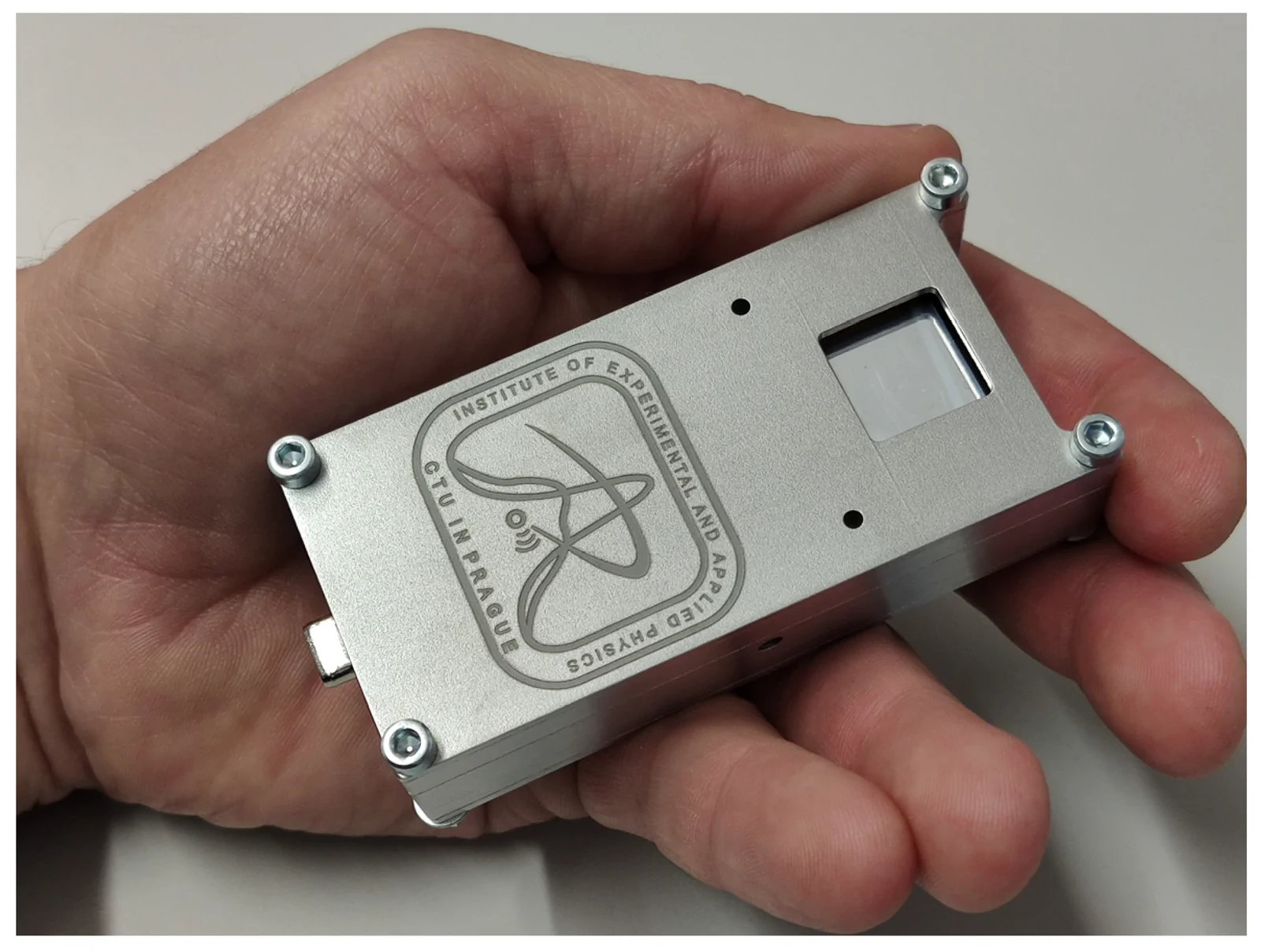
HardPix with TimePix3 sensor visible in the entrance window.

Building on the heritage of the SATRAM project, several follow-up missions and developments have been realized [25,26]. As a direct result of this accumulated experience with space radiation monitoring, IEAP CTU developed the HardPix detector (Figure 2), a next-generation radiation monitor based on the Timepix3 chip. HardPix incorporates onboard data processing and is designed as a versatile, universal radiation monitoring instrument for future space applications. The device has already acquired space heritage through its deployment aboard D-Orbit InOrbit Now (ION) satellites launched in 2023 and 2025, as well as during the Heki ISS mission launched in 2025.

The HardPix instrument comprises two Timepix3 chipboards and an integrated data processing unit. The overall dimensions of the device are 81 × 40 × 22 mm^3^, with a total mass of approximately 130 g. The power consumption during measurement and onboard processing is typically in the range of 3–4 W.

IEAP CTU’s experience in developing Timepix-based neutron spectrometers, combined with the proven space heritage of the HardPix platform, has been consolidated into Neutron HardPix—a proposed neutron spectrometer for space missions, specifically targeted at mapping lunar water deposits aboard rovers, hoppers, and other surface or near-surface vehicles. This study addresses the suitability of Timepix3-based detectors as neutron spectrometers for use on the lunar surface. In the next phase, we will address the modification of the HardPix design for the actual mission.

## 2. Lunar Surface Neutron Count Rate Models

The essential task in assessing the feasibility of Neutron HardPix for mapping lunar water deposits is modeling how expected neutron fluence rates vary depending on the water content of regolith. This modeling was performed by the Institute of Aerospace Medicine at the German Aerospace Center (DLR, www.dlr.de).

### 2.1. Models Used and Input Parameter Assumptions

The radiation environment on the lunar surface caused by primary galactic cosmic radiation (GCR) was calculated for different fractions of water in the regolith with the PLANETOCOSMICS tool [27], which is an application based on the Monte Carlo framework Geant4 [28,29,30]. For the calculation, Geant4 version 11.02 was used and the “Shielding” physics list [31] was selected, which is recommended for applications for neutron transport, in combination with the “EMZ” electromagnetic physics list option [31]. Monte Carlo calculations are a common tool for estimating the radiation environment by measuring the cosmic radiation on the lunar surface: e.g., [32] and references therein.

The primary radiation environment consisted of galactic cosmic radiation nuclei with charge number Z = 1–28 (H to Ni) and energies between 10 MeV/nucleon and 200 GeV/nucleon, as described by the DLR GCR model [33] for solar activity minimum and solar activity maximum conditions. The lunar surface was modeled as a box of regolith with a thickness of 50 m and a side length of 1000 km. The composition of the water-free regolith was taken from the elemental composition of Mare soils (Table 6.3 in [34]) and the corresponding amount of H and O was added for the different fractions of water (Table 1). The primary radiation source was simulated as a point source above the lunar surface, with a cosine-weighting zenith angle for the primary particles. The resulting particle flux was then registered in the plane on top of the regolith. This setup is equivalent to an isotropic primary GCR flux and a point measurement on the lunar surface.

### 2.2. Calculated Spectra

**Figure 3:**
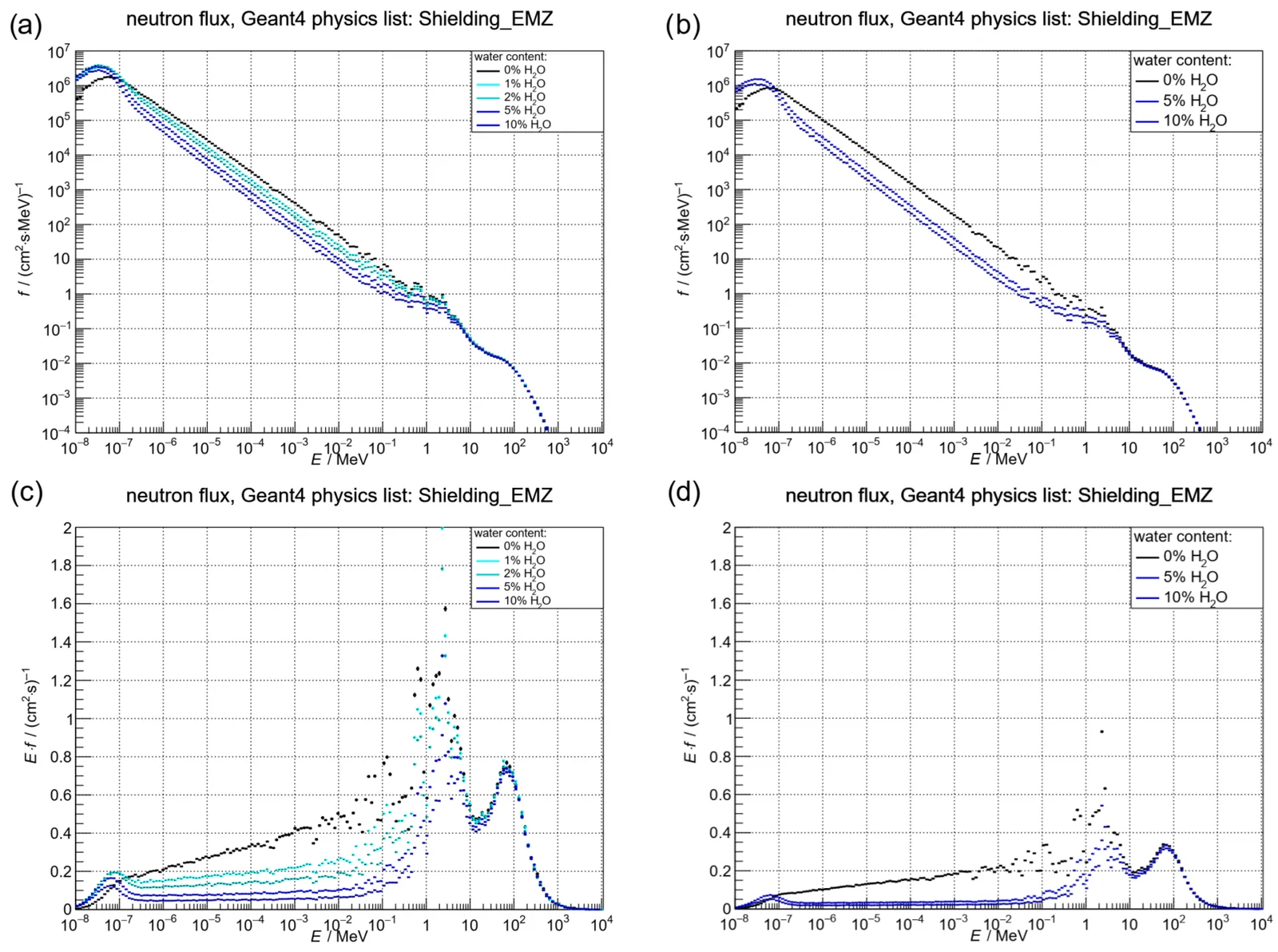
Neutron flux on the lunar surface for different water mass fractions calculated with Geant4/PLANETOCOSMICS for primary GCR nuclei (Z = 1–28) for (**a**) solar minimum conditions, i.e., GCR intensity maximum, and (**b**) solar maximum conditions, i.e., GCR intensity minimum. (**c**,**d**) Contain the identical neutron flux as (**a**,**b**), respectively, but in lethargy representation.

The particles registered on top of the lunar regolith comprised protons, neutrons, photons, e^−^/e^+^, µ^−^/µ^+^, π^−^/π^+^, deuterons, tritons, ^3^He and nuclei from He to Fe. The calculations describe the response of the secondary neutron field as well as the background field of charged and uncharged particles. The calculated neutron spectra for different mass fractions of water (0%, 1%, 2%, 5%, and 10% for solar minimum and 0%, 5%, and 10% for solar maximum) are illustrated in Figure 3 and numerical values of the thermal, epithermal and total neutron fluence rate are given in Table 2 (solar minimum) and Table 3 (solar maximum).

The calculated epithermal and total neutron fluence rates decrease monotonically with increasing water content; the epithermal neutron fluence is reduced by up to approximately 85% for both solar minimum and maximum for the maximum 10% H_2_O mass fraction, and the total neutron fluence by approximately 60%. The calculated thermal neutron fluence increases by approximately 20% for 1% H_2_O mass fraction and is also at an increased level at 2% H_2_O mass fraction. The thermal neutron fluence is reduced by about 30% (solar minimum) and 40% (solar maximum) for the maximum 10% H_2_O mass fraction. Between solar minimum and solar maximum, the neutron fluence decreases by 50% to 60%.

In order to estimate variations in the calculated neutron fluence rates caused by the selection of physics lists in Geant4, a number of physics lists were selected and the model calculations were re-run with an otherwise unchanged setup (solar minimum case). The following additional physics lists were considered [31]:

- QGSP_INCLXX_HP_EMZ (Quark-Gluon String + Precompound + Liège Intra-Nuclear Cascade + High Precision neutron package + Electromagnetic Option 4)
- FTFP_INCLXX_HP_EMZ (Fritiof string + Precompound + Liège Intra-Nuclear Cascade + High Precision neutron package + Electromagnetic Option 4)
- FTFP_BERT_HP_EMZ (Fritiof string + Precompound + Bertini Intranuclear Cascade + High Precision neutron package + Electromagnetic Option 4)

For the QGSP_INCLXX_HP_EMZ, all five values for the water content were modeled, for the other two only the minimum (0% H_2_O) and maximum (10% H_2_O) were used. Table A1 (Appendix A) contains the results for the fluence rates of thermal and epithermal neutrons and the total neutron fluence rate. The relative differences with respect to the previous physics list Shielding_EMZ are summarized in Table A2 (Appendix A). The two physics lists using the INCLXX model in the intermediate energy range (20 MeV to 20 GeV) are very similar to within a few percent, and are between 25% to 33% lower compared with Shielding_EMZ and FTFP_BERT_HP. It should be noted that the latter two both apply the BERT model for the intermediate energy range. The high-precision (HP) model is part of all the listed physics lists for the neutron energy range below 20 MeV.

To investigate the expected variation in neutron fluence rate for an alternative choice of regolith, a composition which is considered typical of the lunar highlands was also applied (Ferroan ANorthosite, FAN, Table A3 in Appendix A).

Using the FAN regolith composition, the neutron fluence rates were calculated with the Shielding_EMZ physics list for solar minimum conditions (Table 4). For low water content, the thermal neutron fluence is considerably higher (~70%) than previously calculated for the mare regolith. These differences disappear with increasing water content and are reduced to 2% for 10% water content. The epithermal neutron fluence is only slightly affected by the regolith composition and a few percent lower for the FAN regolith compared to the mare regolith.

**Figure 4:**
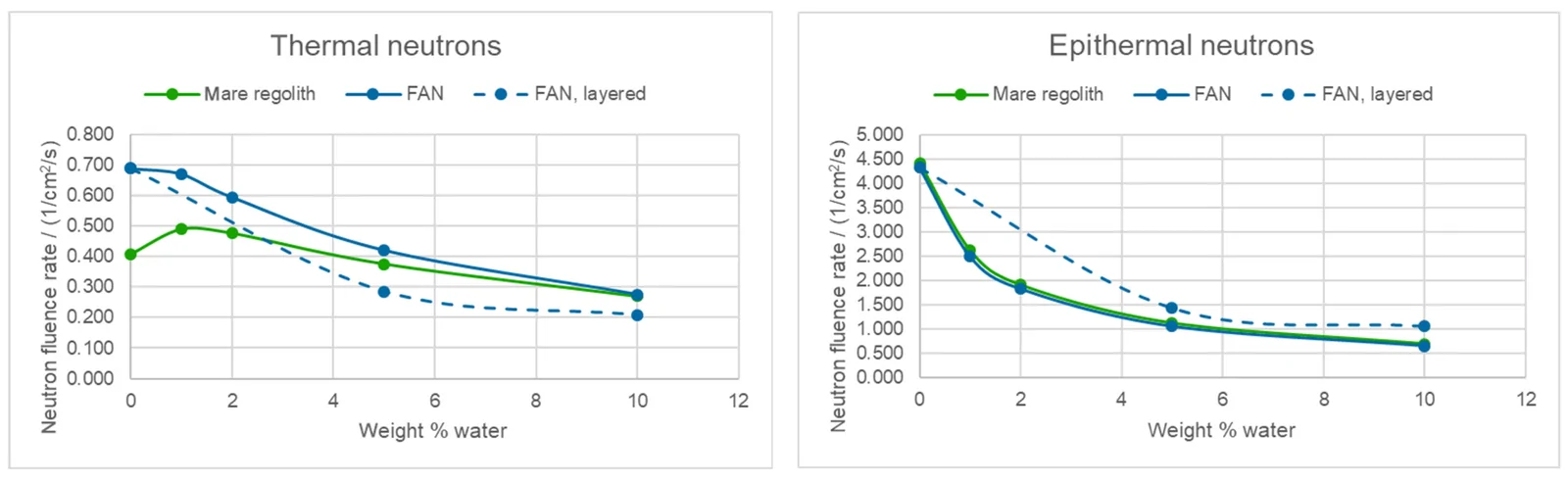
Thermal (**left**) and epithermal (**right**) neutron fluence rates calculated for average mare regolith and FAN regolith, dependent on the water content of the regolith. In the layered setup, the wet regolith is covered with a 50 g/cm^2^ layer of dry regolith.

In a layered setup, the impact of a potentially dry layer above the wet layer was investigated. The dry layer corresponded to 50 g/cm^2^ (~16.7 cm at a density of 3 g/cm^3^) FAN regolith on top of the wet layer of FAN regolith with either 5% or 10% water content. Using the layered geometry for 5% and 10% water content reduced the thermal neutron fluence by about 30% and 20% and increased the epithermal neutron fluence by about 40% and 60%, respectively (Table 4, Figure 4).

### 2.3. Comparison to Data in the Literature

The expected signal in the Lunar Prospector Neutron Spectrometer to varying amounts of water in the lunar regolith was estimated by [6,35,36]. Although most of their results include a detector response function and a transport function to the orbit and thus represent the detector count rate and not the thermal and epithermal neutron fluence on the lunar surface, a qualitative comparison can be made. For FAN regolith, ref. [35] calculated a maximum increase of 70% in thermal neutron counts with increasing water content up to a few percent and a steep decrease at higher values. A similar but less pronounced pattern shows in the calculations for the mare regolith (Table 2, Figure 4), with a maximum increase of about 20% at 1% water content. This pattern is, however, absent in the calculations for the FAN regolith (Table 4, Figure 4). The reason for this discrepancy is unknown but could be caused by different energy ranges or detector sensitivity and orbital transport function used by [35].

For epithermal count rates, ref. [35] calculated a monotonically decreasing count to about 70% of the initial count at 1% water content and about 20% of the initial count at 10% water. This is comparable to the results for FAN regolith (Table 4, Figure 4), which show a reduction to about 60% and 15% of the initial fluence rate at 1% and 10% water content, respectively.

Ref. [35] also presented in their Figure 1 differential neutron spectra on the lunar surface calculated for a number of regolith compositions. The peak neutron flux in the thermal energy range was between 5∙10^5^ cm^−2^s^−1^MeV^−1^ and 5∙10^6^ cm^−2^s^−1^MeV^−1^ at approximately 0.5∙10^−7^ MeV to 1∙10^−7^ MeV, which is in agreement with the results shown in Figure 3.

## 3. Neutron HardPix Converter Design

Semiconductor neutron detectors offer a compact solution for neutron detection and imaging applications. Owing to the absence of electric charge, neutrons cannot be detected directly through conventional ionization mechanisms; therefore, their detection in semiconductor devices generally relies on secondary effects. A common approach involves the deposition of neutron-reactive films onto semiconductor charged-particle detectors, where neutron capture reactions generate charged particles that can subsequently be detected.

### 3.1. Basic Concept of Thermal Neutron Detection

Among the neutron interactions most frequently exploited in thermal neutron detection are the ^10^B(n,α)^7^Li reaction and the ^6^Li(n,α)^3^H reaction (see [37], and the discussion therein). Thin-film neutron detectors typically consist of semiconductor detectors coated with one or more neutron-reactive conversion layers. Neutron absorption within the converter produces charged particles emitted in opposite directions, one of which may penetrate the semiconductor and deposit its energy, thereby enabling neutron detection.

### 3.2. Converter Material

The ^10^B(n,α)^7^Li reaction has a higher interaction probability than ^6^Li(n,α)^3^H for neutron energies below about 100 keV because of its larger absorption cross-section. However, the higher kinetic energies of the charged particles produced in the ^6^Li(n,α)^3^H reaction improve their detectability. This advantage is especially important in mixed-radiation environments, such as space, where neutron-induced events must be distinguished reliably from background. In Timepix detectors, ^6^Li(n,α)^3^H reaction products generate well-defined track signatures that support effective event discrimination. Therefore, ^6^Li-based conversion layers are considered particularly suitable for thermal and epithermal neutron detection in the Neutron HardPix instrument. While pure ^6^Li converters may provide thermal neutron detection efficiencies exceeding about 11% [37], their high chemical reactivity complicates fabrication and limits long-term stability. As a result, lithium fluoride (LiF), which is chemically more stable, is more commonly used in practice.

### 3.3. Converter Thickness

Neutron capture may take place at any position within the conversion layer. The charged reaction products generated in the neutron-reactive film lose part of their energy while traversing the converter material before they can reach the semiconductor sensor. This energy loss reduces the amount of energy ultimately deposited in the detector and thus affects the amplitude of the measurable signal.

The finite stopping power of the converter material sets a practical upper limit on the useful thickness of the conversion layer. Although a thicker layer increases the probability of neutron capture, this improvement is only beneficial up to the point at which the reaction products can still escape the converter and enter the semiconductor. Beyond that thickness, the charged particles are fully absorbed within the conversion layer and no longer contribute to detectable events. Additional increases in converter thickness therefore do not improve the detector response.

**Figure 5:**
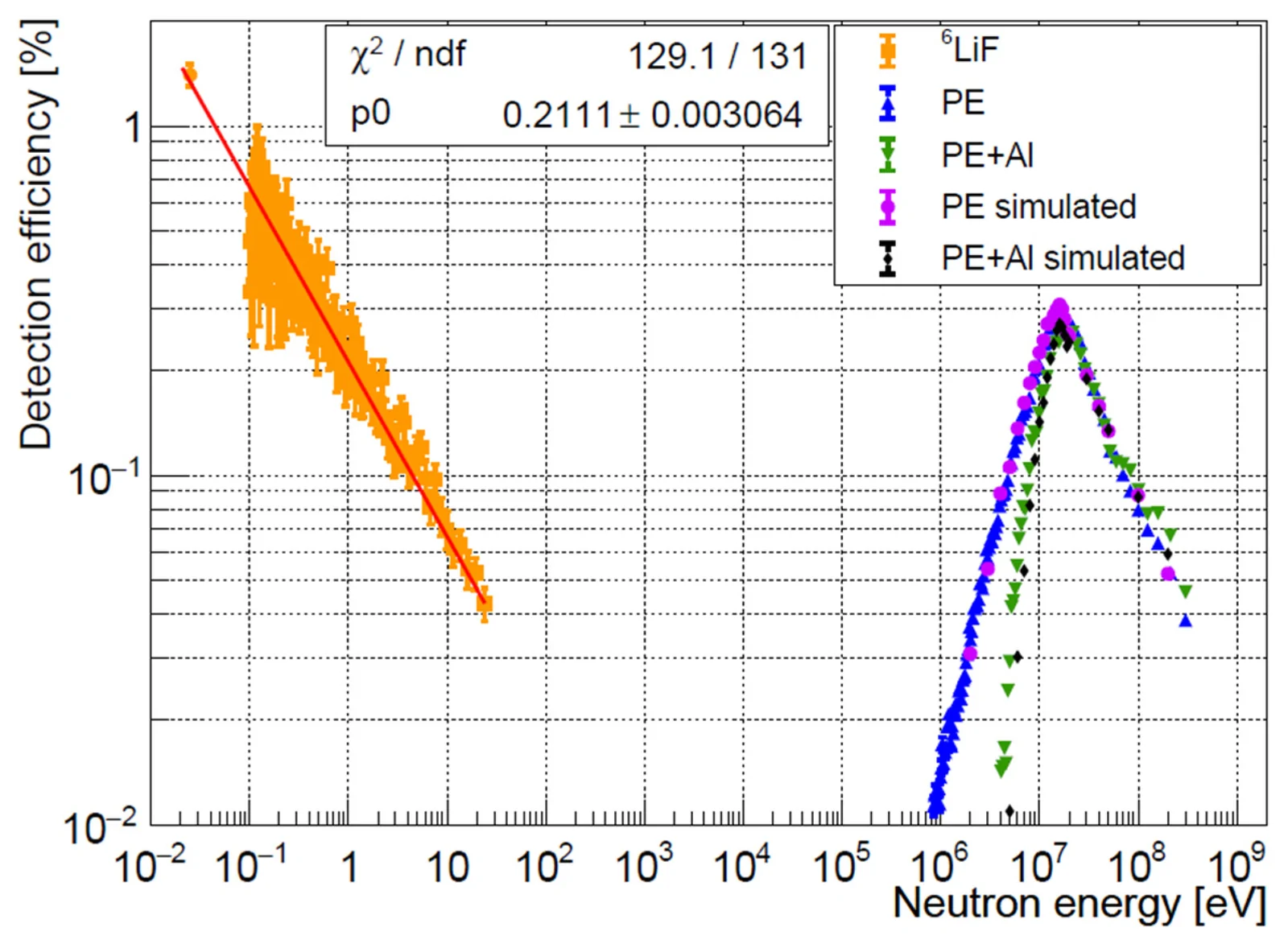
Thermal and fast neutron detection efficiencies of LiF and PE converter layers used with the Timepix3 detector (from [38]) Red line represents a fit to the measured data.

The IEAP CTU has extensive experience with LiF converter films applied to Timepix sensors [38,39] and used at the Czech Metrology Institute (CMI, cmi.gov.cz), the Los Alamos Neutron Science Center (LANSCE, lansce.lanl.gov), ATLAS LHC and other facilities. However, we have never had to worry about very low fluence rates, so detection efficiency was not an issue and we opted for a very thin LiF layer of about 5–6 µm, providing thermal neutron detection efficiency of around 1% (Figure 5).

McGregor et al. [37] claimed maximum efficiency of the LiF converter layer to be close to 4.5% using thickness of around 25 µm on a silicon diode. To confirm this efficiency with our pixelated sensor and perform a trade-off between detection efficiency and signal to noise ratio, we tested several thicknesses of LiF film and describe the results in the next chapter.

### 3.4. Epithermal Neutrons Conversion Layer

As discussed in the previous section, Li absorption cross section follows a 1/v dependence. Therefore, the energy-dependent efficiency of the ^6^LiF decreases with energy as *ε* ∼ $\frac{1}{\sqrt{En}}$ (see Figure 5). This means that an LiF converter is sensitive to epithermal neutrons as well, though with significantly reduced efficiency compared to thermal neutrons. In order to precisely separate the fluence rates of thermal and epithermal neutrons, and to assess whether Neutron HardPix can use also epithermal neutron fluence rate to map water deposits, we included a cut-off filter in the form of Cadmium foil.

This is a similar method to one used by gas-proportional counters with two tubes, such as LEND, where one tube is equipped with Cd foil that blocks thermal neutrons, making this tube sensitive to epithermal neutrons only. The second tube detects both thermal and epithermal energies. Thermal neutron fluence rate is then obtained by deducting the epithermal rate from the total one. We used an identical method and wrapped the thermal neutron setup in Cd foil to obtain epithermal neutron fluence rate and assess the feasibility of such solution for Neutron HardPix.

### 3.5. Fast-Neutron Conversion Layer

Fast-neutron conversion layers have a form of a thin (~1 mm) polyethylene (PE) layer in front of the Timepix3 sensor. Fast neutrons are detected by recoil protons created by fast neutron interactions with the hydrogenous material.

**Figure 6:**
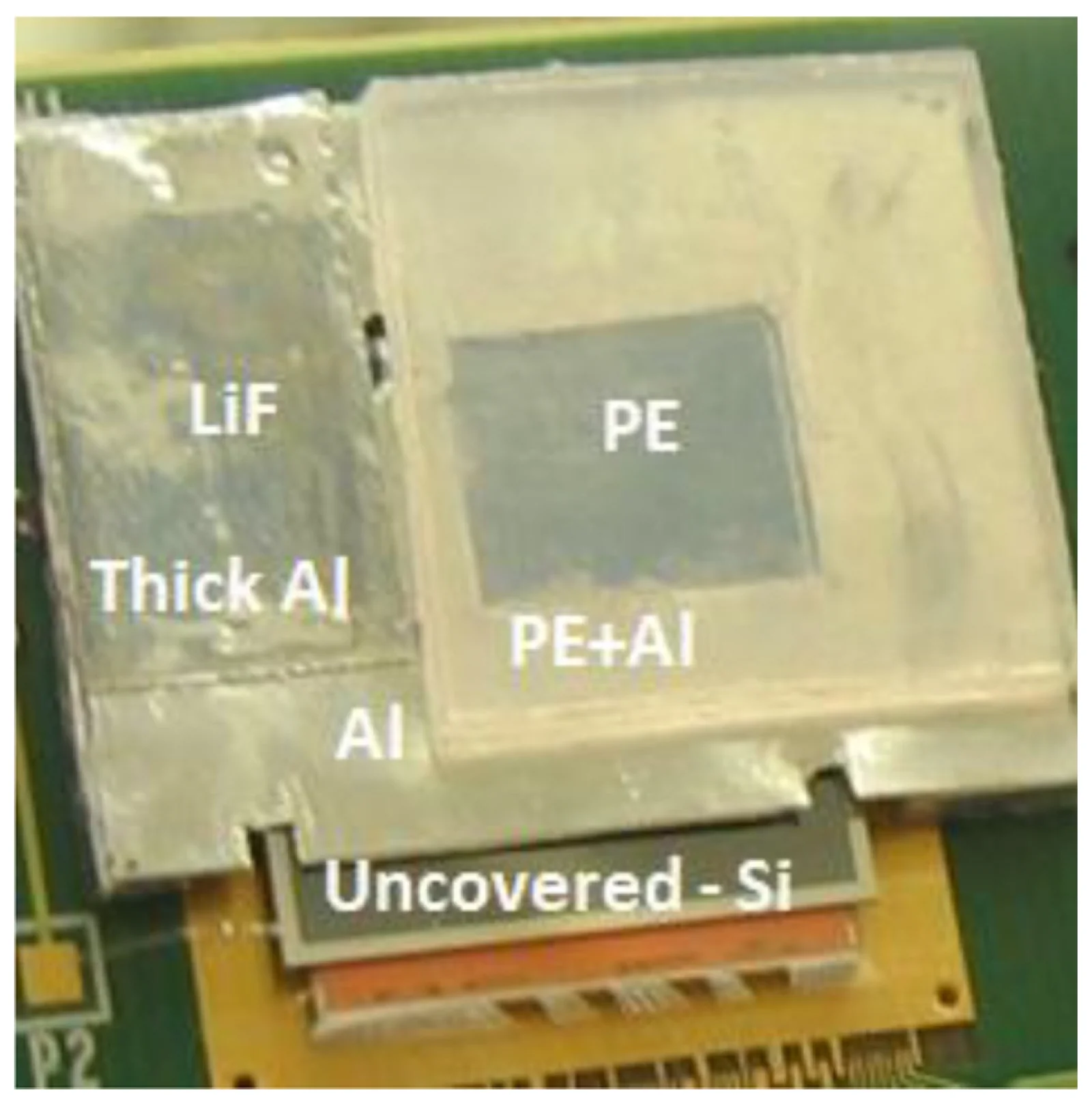
Example of the PE conversion mask on the Timepix detector.

We experimented with PE converters for as long as with LiF (Figure 6), running a network of neutron detectors in the ATLAS LHC cavern and measuring at the LANSCE facility, where we obtained a peak detection efficiency for fast neutrons of 0.3% at ~16 MeV (see Figure 5).

## 4. Neutron HardPix Breadboard Efficiency Measurements

### 4.1. Measurements Using AmBe Source at Van De Graaff Accelerator

The laboratory of the Van de Graaff (VdG, https://aladdin.utef.cvut.cz/projekty/vdg, accessed 1 July 2026) accelerator is devoted to basic and applied research in experimental nuclear physics. Besides serving as a source of light ions the facility serves as a tuneable source of mono-energetic neutrons in a relatively wide energy range (100 keV–19 MeV).

**Figure 7:**
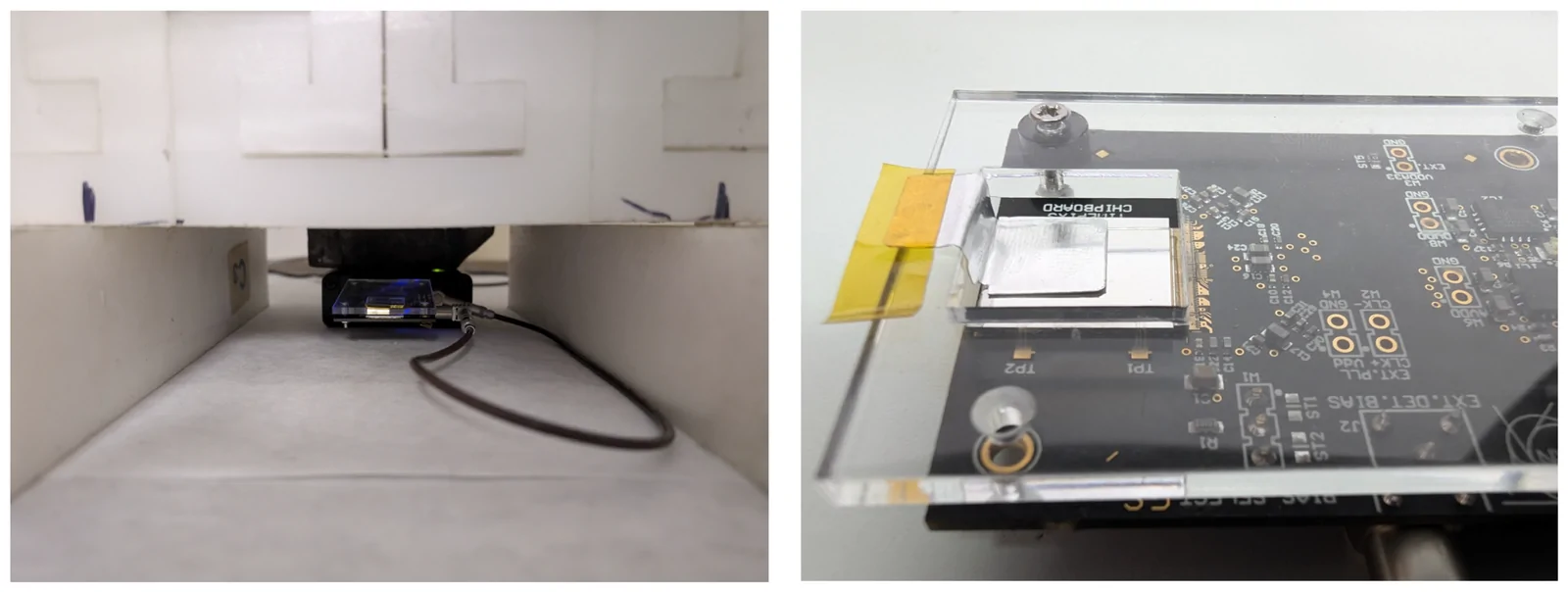
Pictures of the experimental setup in the AmBe measurement. (**Left**) Timepix3 detector located under a layer of white polyethylene bricks that moderate the neutrons from the AmBe source. The AmBe source itself is not visible. (**Right**) A thin 100 μm thick piece of aluminium foil placed on top of the Timepix3 sensor. The bottom side of the aluminium foil carries the LiF converter layer.

Besides the accelerator, the facility is equipped with an Americium-Beryllium (AmBe) source, producing a continuous spectrum of neutrons with energies in the range of ∼0.5–10 MeV. To slow down the neutrons, the AmBe source was inserted inside a hole in a polyethylene (PE) box with 8 cm of PE between the source and the Timepix3 detector (Figure 7). First, we made a comparison between four manufactured LiF layers of different thicknesses: 6.09 mg cm^−2^, 4.88 mg cm^−2^, 3.08 mg cm^−2^, and 1.44 mg cm^−2^, which correspond to approximately 6, 12, 18 and 24 µm. These LiF layers were spray-coated onto aluminium foils which are interchangeable so that an identical Timepix3 chipboard can be used for testing different layers.

Foils with LiF films have an area of 1 × 1 cm^2^ and cover half of the Timepix3 sensor area. An uncovered region (Si) is used for background subtraction from high-energy transfer particles, leaving similar signatures in the sensor as conversion products. In this case, the background is given by the non-neutron field as well as neutron interactions in silicon, such as neutron elastic and inelastic scattering, ^28^Si(n,α)^25^Mg-reactions, ^28^Si(n,p)^28^Al reactions, more complex processes, and spallation reactions with thresholds of 2.75 MeV, 4 MeV, and ≥20 MeV, respectively.

The lithium isotopic composition contained 96% ^6^Li. The LiF layer was placed as close above the sensor as possible (Figure 7) so that the tritium and helium nuclei need to cross the minimum distance through the air. Although the exact neutron field was not known, it was the same for all four tested LiF layers, therefore allowing us to make a relative comparison.

**Figure 8:**
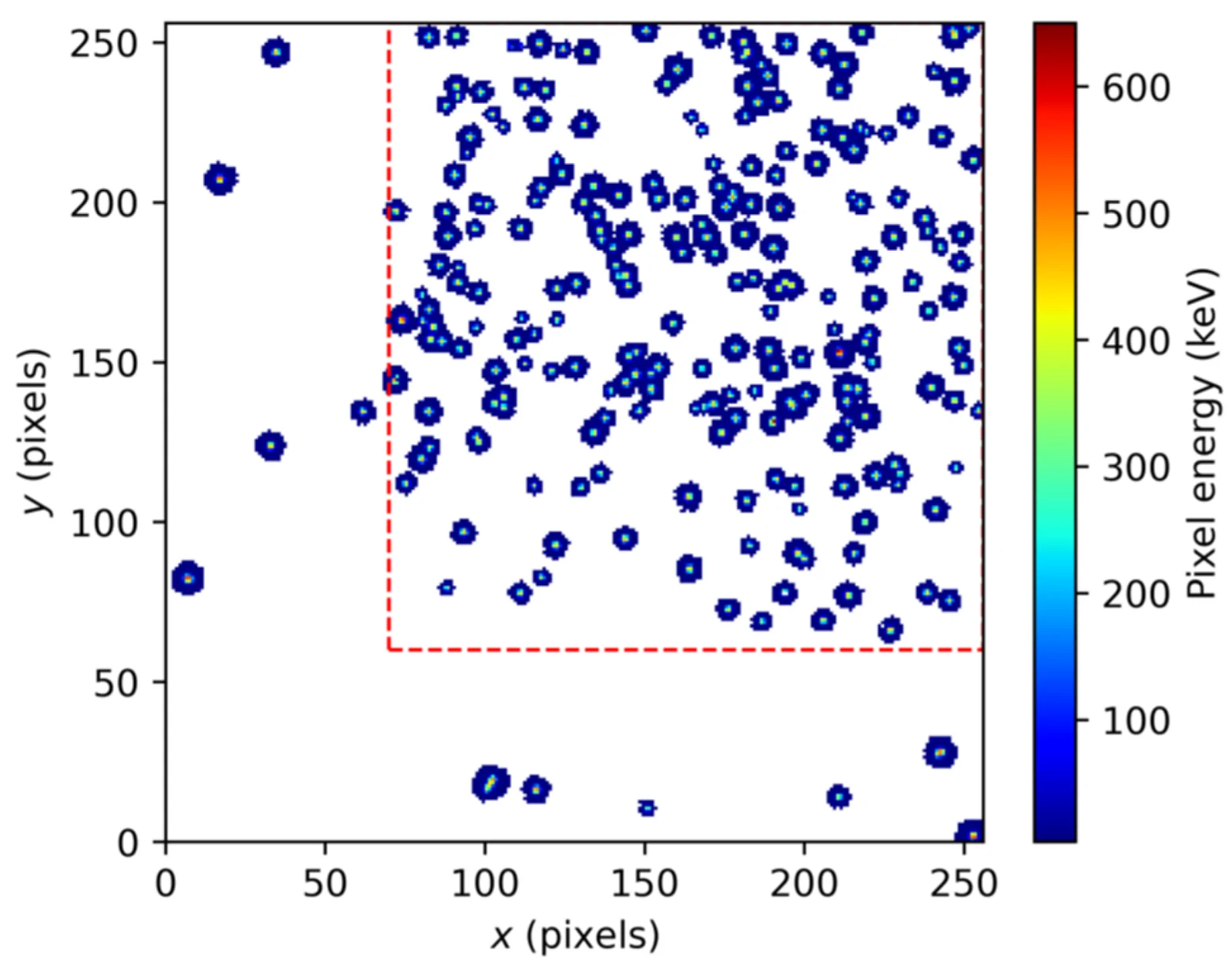
Clusters of shape “heavy blob” in the 256 × 256-pixel matrix of Timepix3. The color scale shows energy in keV deposited in pixels. The top right region inside the red rectangle shows higher density of heavy blobs—it is where the LiF converter was located, and most of these heavy blobs were created by tritium and helium nuclei from the conversion. Heavy blobs in the region without the LiF converter come from the background (e.g., alpha particles from radon gas).

Tritium and helium nuclei with energies in the order of ~2 MeV from the ^6^Li(n,α)^3^H reaction create clusters of shape called “heavy blobs” in Timepix3 (Figure 8). A “heavy blob” cluster satisfies all of these conditions:

- It has 4 or more inner pixels, i.e. pixels not lying on the border of the cluster.
- Its ratio of the number of inner pixels to the number of border pixels is 0.5 or more.
- The cluster is roughly of a round shape—maximum distance between any pair of pixels is smaller than 1.2× the diameter of a hypothetical circular cluster of the same area.

**Figure 9:**
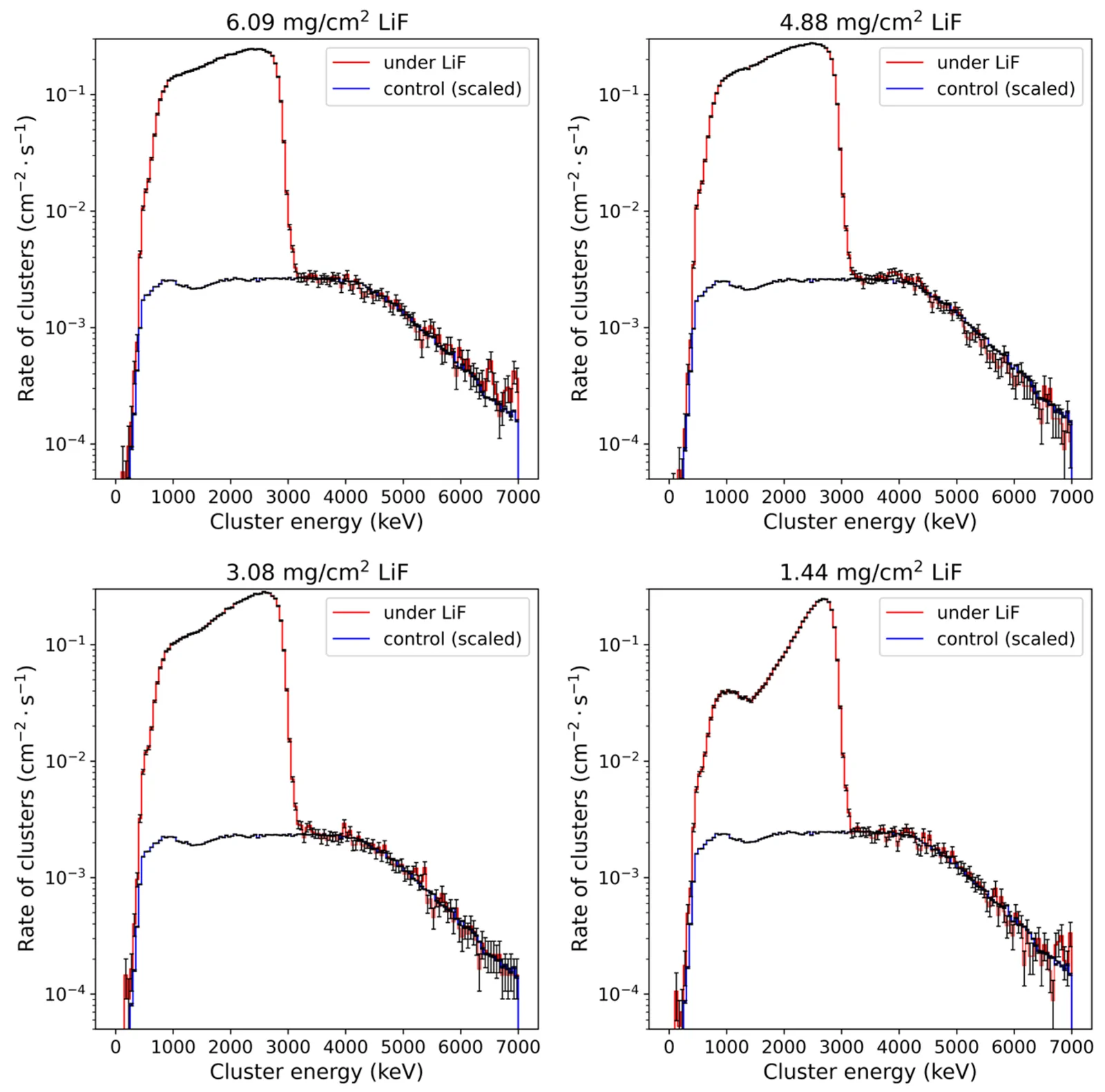
Energy spectrum of heavy blobs under the LiF region (red) compared to the energy spectrum of heavy blobs in the control region (blue). The blue spectrum was scaled by a constant to match the red spectrum at high energies. Black markings represent errorbars of the datapoints.

The region of the sensor under the area covered with LiF contains these heavy blobs as well as background heavy blobs and other ionizing particles. To remove background, we subtract heavy blobs in the control region without LiF and filter out particles (e.g., electrons and minimum ionizing particles), which create clusters of different shapes. Since the maximum detectable energy of the ^6^Li + n products is 2.73 MeV for the tritium nucleus, the deposited energy spectra of heavy blobs under LiF and in the control region have the same shape at higher energies. The spectra for the four different LiF layer thicknesses are shown in Figure 9.

The difference in spectra between different LiF converter thicknesses is clearly visible and demonstrates the effect of conversion products losing energy while traversing the converter layer itself, broadening the spectrum. While the thinnest layer has the lowest detection efficiency (as discussed later) it has a sharp peak in the energy spectrum which can be exploited in filtering the heavy blobs coming from the neutron conversion from the background with similar cluster shape.

**Figure 10:**
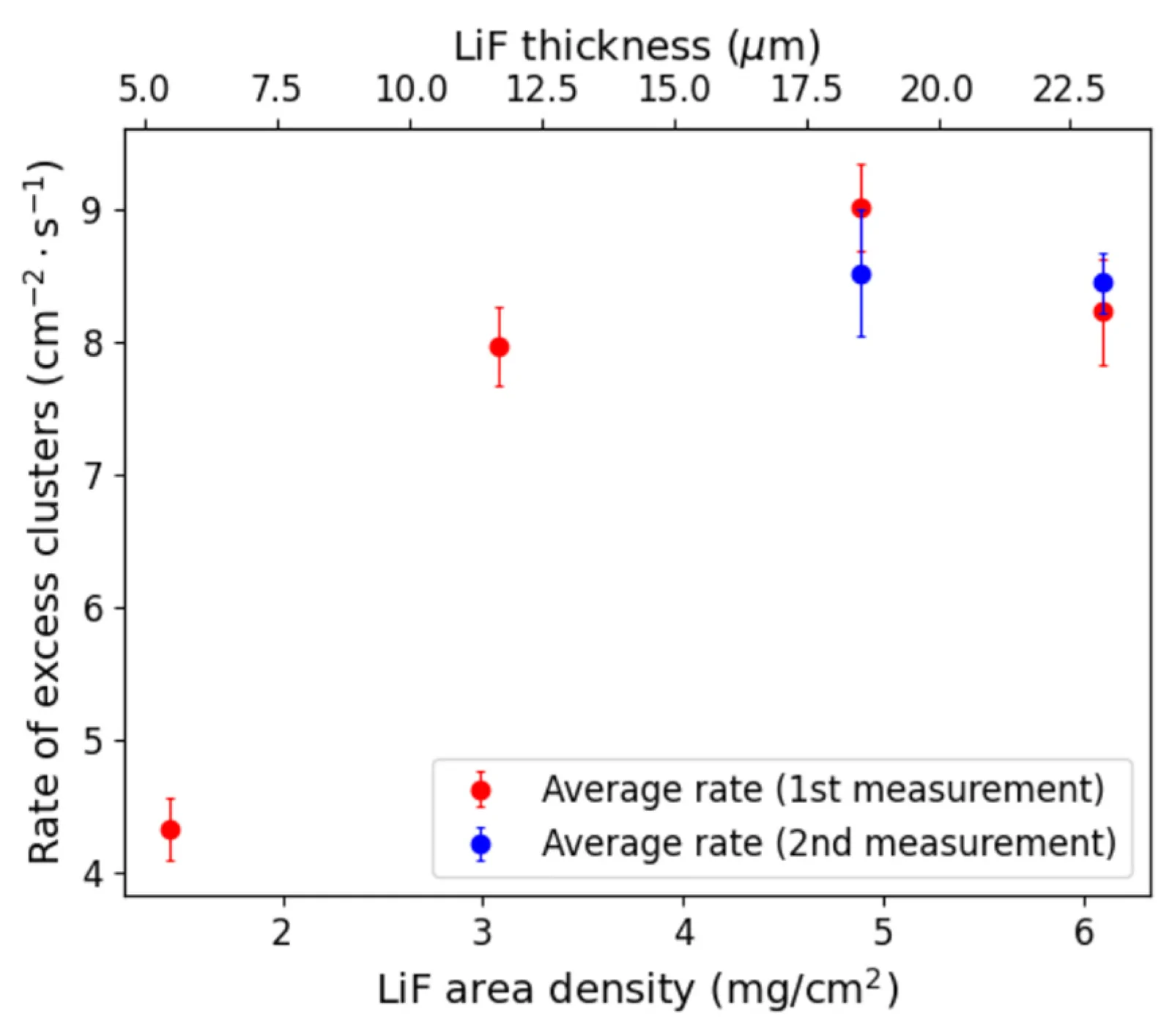
Comparison of the rate of tritium and helium products of 6Li + n interactions under LiF layers of different thicknesses. Red data points show results from measurements taken at the beginning of October 2024. The blue data points are from the end of October 2024, when we repeated a few measurements to prove that we can consistently reproduce the same conditions. Errors were estimated by dividing the sensor area under the LiF converter into nine distinct areas of roughly the same size, calculating the rate of tritium and helium heavy blobs in each of them and calculating the standard deviation of these nine rates. The 4.88 mg cm^−2^ LiF layer had the highest rate of tritium and helium nuclei (8.85 ± 0.27) cm^−2^ s^−1^, as shown by the weighted average of the two measurements.

By integrating the spectrum with the energy we get the total rate of tritium and helium nuclei, giving us the relative detection efficiency of each layer. Figure 10 shows that the 4.88 mg cm^−2^ LiF layer was the most efficient of the four layers tested, achieving the rate of tritium and helium nuclei (8.85 ± 0.27) cm^−2^ s^−1^. The thicker layer had a similar rate, which is in agreement with the McGregor [37] sensitivity curve where the rate enters a slowly declining plateau after an initial rise due to conversion products not penetrating the LiF layer from larger distances from the sensor.

The different rate of 4.88 mg cm^−2^ LiF layer between the first and second measurement was caused most probably by the different proximity of the LiF converter layer to the sensor. Aluminum foils with different converter thicknesses were attached by hand and even a few microns of air gap difference will have a measurable effect on the remaining energy of tritons and alphas. Another difference from the idealistic models is that the aluminum conductive layer on top of the Timepix3 chip required bias, which is discussed later.

After finding that the 4.88 mg cm^−2^-thick LiF layer is the most efficient for neutron conversion, we decided to perform further experiments only with this layer. First, we tested the effect of cadmium shielding, which captures thermal neutrons but allows more energetic neutrons to pass.

**Figure 11:**
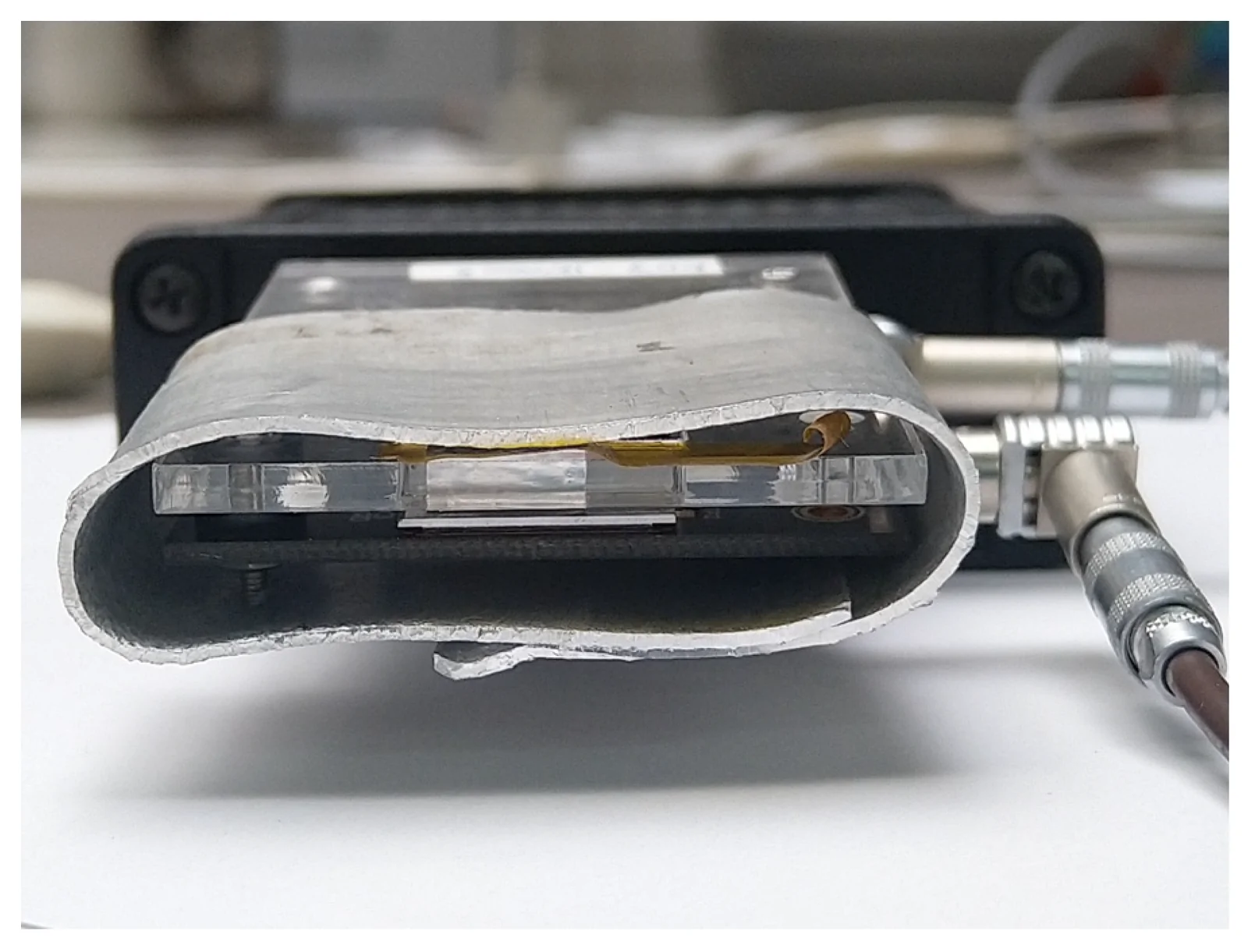
Timepix3 detector with 1 mm-thick cadmium metal shielding wrapped around the sensor. There were openings on the sides of the sensor, but the sleeve had ∼1 cm excess on both sides of the openings.

Figure 11 shows a side view of a Timepix3 detector with a cadmium metal sleeve wrapped around it. Although there were openings in the cadmium shielding, the excess of the cadmium shielding was ∼1 cm on both sides of the sensor with openings. Some thermal neutrons may have diffused through these openings and reached the LiF converter, but we consider this number negligible given that the AmBe source was located in front of the sensor. The measured rate of heavy blobs with this shielding under the area with the LiF convertor was (0.633 ± 0.027) cm^−2^ s^−1^. We can compare this to the (8.85 ± 0.27) cm^−2^ s^−1^ rate from the previous measurement and calculate that after we subtract the epithermal neutron rate behind Cadmium shield, the thermal neutrons accounted for (8.22 ± 0.27) cm^−2^ s^−1^ hits.

A small amount of uncertainty comes from the fact that while most of the thermal neutrons came from the polyethylene box, some were thermalized elsewhere, for example in the wooden table on which the experiment sits. Some helium and tritium nuclei were possibly created directly by the fast neutrons (the cross section of ^6^Li + n → ^3^H + ^4^He reaction for fast neutrons is orders of magnitude smaller compared to slow neutrons, but non-zero).

Next, we performed measurements without the PE moderator to see the response in a field dominated by fast neutrons, which is a scenario expected on lunar surface. This gives us an upper estimate of how many heavy blobs in previous measurements were created by neutrons other than those moderated by the polyethylene box. The measured rates of heavy blobs were (0.111 ± 0.010) cm^−2^ s^−1^ under LiF and (0.028 ± 0.010) cm^−2^ s^−1^ under LiF covered with Cadmium shielding, which represent the systematic error of the thermal and epithermal neutron measurements with the LiF converter.

This exercise represents a realistic scenario during a lunar rover mission, where we can expect to detect not only thermal and epithermal neutrons moderated in the lunar regolith but also those moderated by the materials of the rover. This background can be mitigated by placing the detectors at the bottom of the spacecraft as close to the surface as possible though not entirely eliminated.

### 4.2. Calibration Using AmBe Source at the Czech Metrology Institute

**Figure 12:**
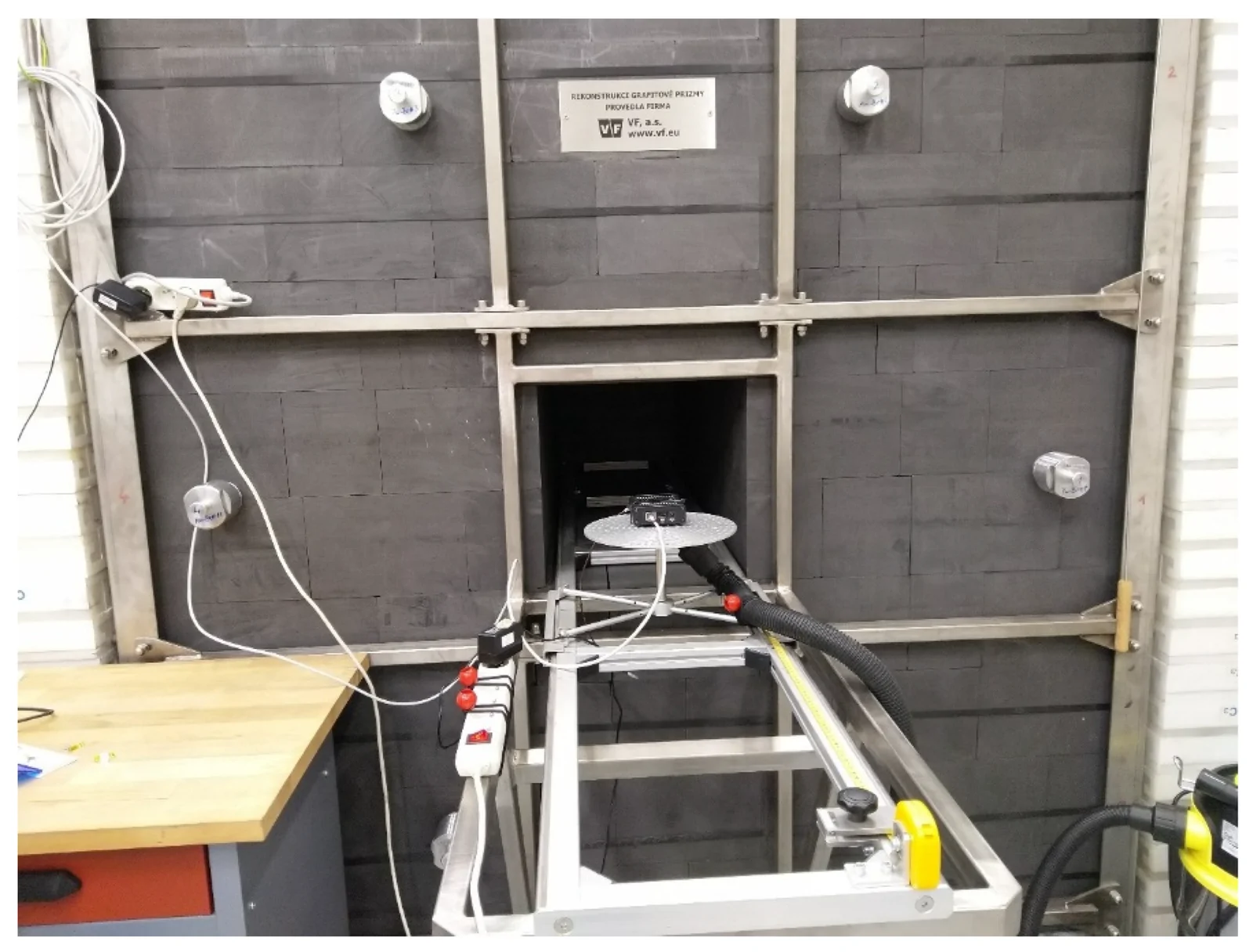
A graphite pile at CMI with 6 AmBe sources inserted around the detector in the middle.

The Czech Metrology Institute (CMI) in Prague is equipped with the calibrated field of slow neutrons. It is created by inserting six AmBe sources in a graphite pile (Figure 12). Graphite serves as a moderator and in its center the neutron field is close to isotropic, with a fluence rate of (2.896 ± 0.035) × 10^4^ cm^−2^ s^−1^, of which 92% of neutrons are below 0.5 eV kinetic energy; i.e., thermal. The fluence rate is defined as the number of neutrons hitting a sphere divided by the cross-sectional area of the sphere. Therefore, the number of neutrons hitting a flat square area (such as the LiF converter) from all sides is half of the fluence rate (1.448 ± 0.018) × 10^4^ cm^−2^ s^−1^.

**Figure 13:**
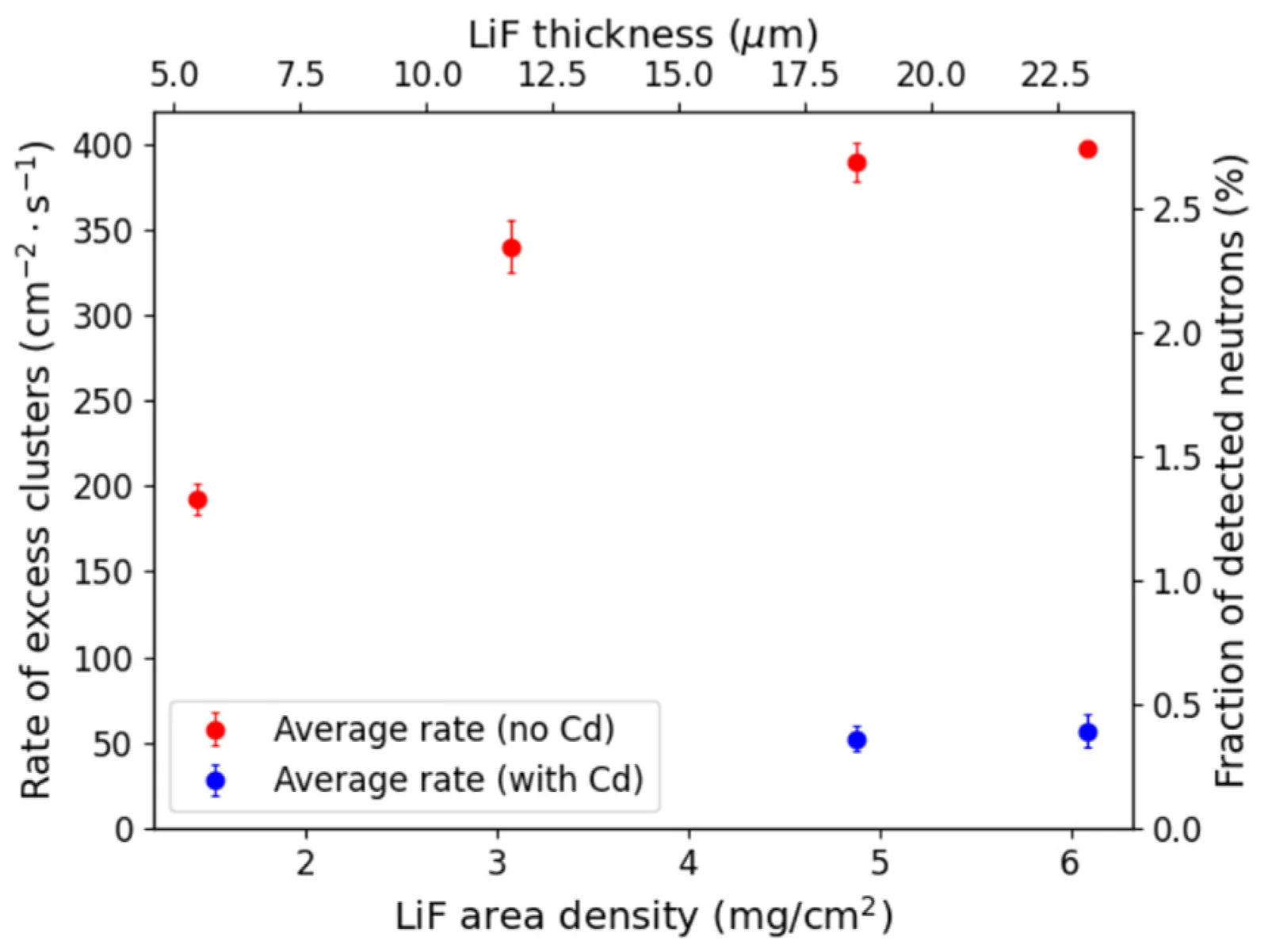
The plot of the average rate of heavy clusters under the LiF converter as a function of converter thickness measured at CMI. The left vertical axis shows the absolute flux of heavy blobs, while the right axis shows the flux as a percentage of the total neutron fluence rate (1.448 ± 0.018) × 10^4^ cm^−2^ s^−1^. The blue point measurements with cadmium shielding were likely contaminated by some thermal neutrons that passed through the openings in the shielding. The blue points are thus only upper estimates. The error bars were calculated in the same way as in Figure 10.

Measurements with all four LiF layers of thicknesses 6.09 mg cm^−2^, 4.88 mg cm^−2^, 3.08 mg cm^−2^, and 1.44 mg cm^−2^ were repeated in this neutron field. Results are shown in Figure 13. The background at CMI was negligibly low. The most efficient convertor, with the highest heavy blob count rate, was the 6.09 mg cm^−2^ one with a rate of (397 ± 4) cm^−2^ s^−1^. Within error bars, it was equaled by the 4.88 mg cm^−2^ converter, with (389 ± 11) cm^−2^ s^−1^ heavy blobs under LiF. Dividing by the neutron fluence rate (1.448 ± 0.018) × 10^4^ cm^−2^ s^−1^, we arrive at efficiencies of detection of (2.74 ± 0.04)% and (2.68 ± 0.08)% for the 6.09 mg cm^−2^ and 4.88 mg cm^−2^ converters, respectively. This is a calculation assuming a constant efficiency over all energies produced at CMI. Because efficiency depends on neutron kinetic energy according to the relationship ε ∝ E^−1/2^, peak efficiency for thermal neutrons at low energies will be higher.

**Figure 14:**
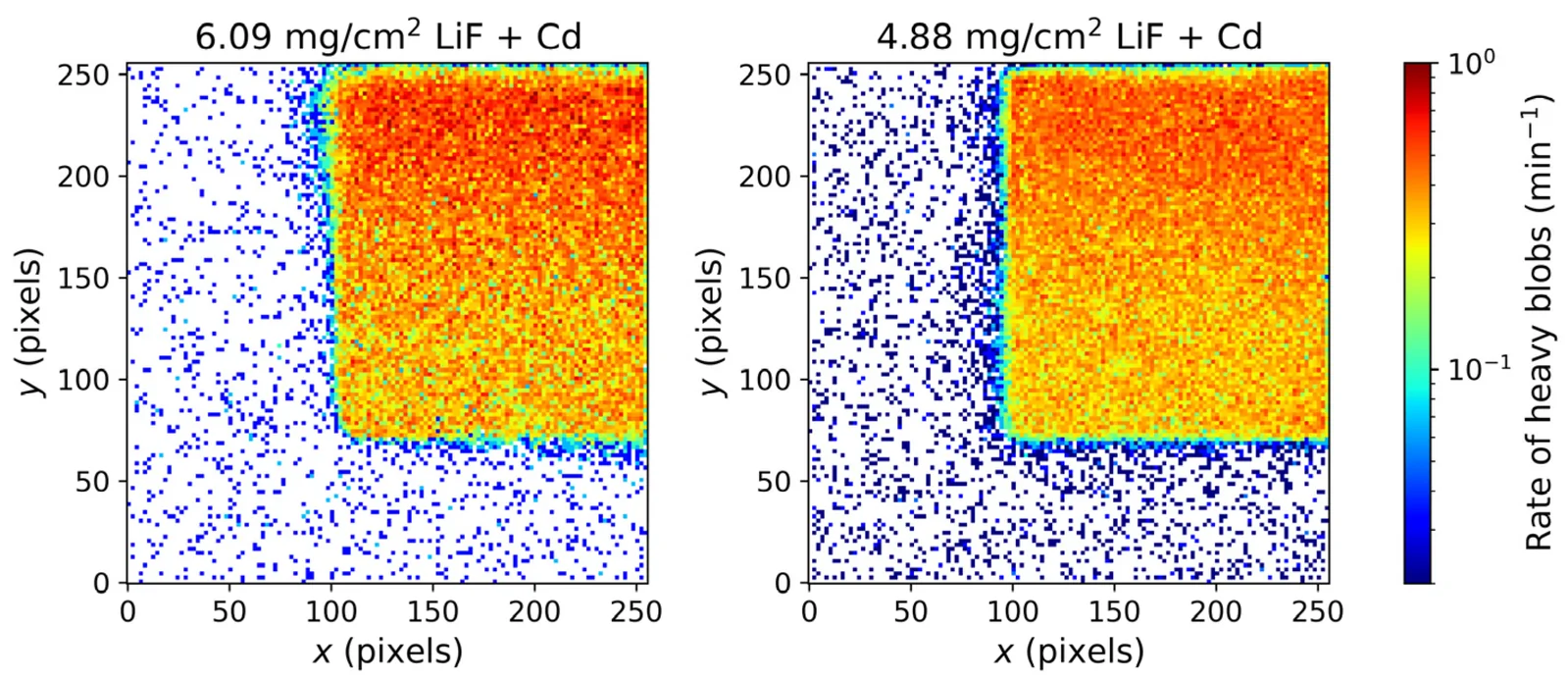
Density of heavy blobs in the measurement at CMI with the cadmium shielding. The LiF converter was placed in the upper-right corner of the sensor. The density of heavy blobs under LiF is ∼1.5 higher in the upmost region compared to the lowest region.

For 6.09 mg cm^−2^ and 4.88 mg cm^−2^ converters, we also performed measurements with the cadmium shielding. However, the rate of heavy blobs under the LiF converter was not uniform (see Figure 14). These were likely thermal neutrons diffusing through the side opening in the cadmium shielding (see Figure 11). We did not see such an effect in the measurements performed at the VdG, probably due to VdG sources being placed in front of the sensor vs. the CMI neutron field being isotropic with thermal neutrons coming also from the sides.

This was the first reason why we were unable to reliably obtain detector efficiency for epithermal neutrons. The second was the fact that CMI does not provide the fluence rate for epithermal neutrons, only for thermal ones, and while it is thought that 92% of neutrons are thermal, the exact portion of epithermal ones in the remaining 8% is unknown. Nevertheless, we can estimate it using fact that the LiF efficiency is directly proportional to E_k_^−1/2^.

### 4.3. Fast Neutrons Calibration at LANSCE Facility

We performed measurements at the VdG with the Timepix3 sensor and polyethylene converter which showed an excess of heavy blobs under the converter. However, given that we were not familiar with the neutron field at the VdG facility and we had no other measurements to compare this relative measurement to, we relied on our previous measurement with Timepix3 at the Weapons Neutron Research (WNR) facility of the Los Alamos Neutron Science Center (LANSCE). The fast-neutron converter was a ∼1 mm-thick polyethylene layer in front of the Timepix3 sensor, serving the purpose of detecting recoil protons generated from fast neutron interactions with the hydrogen-rich material. Details of the setup at LANSCE are described in [38].

Neutron detection efficiency is shown as a function of the neutron energy in Figure 5. An increased response below the PE layer becomes visible above a neutron energy of 1 MeV. With increasing neutron energy, the range of recoil protons increases, so that the efficiently used thickness of the converter increases. At higher energy, however, the recoils receiving large amounts of energy from the neutrons become penetrating, leaving only part of their energy in the silicon sensor until, eventually, the energy is not sufficient to create the required neutron signature of a heavy blob or track. Thus, maximal efficiency for detection of fast neutrons of *ε*_max_ = 0.3% was found at ∼16 MeV.

### 4.4. Calibration Summary

We calibrated the Neutron HardPix detector at different facilities and obtained thermal neutron detection efficiency of 2.7% average. While McGregor et al. [37] claims maximum efficiency close to 4.5% in an ideal scenario, there are some aspects of our measurement which will reduce the efficiency to values observed by us. The first is a small air gap between the Timepix3 sensor and the converter layer where Helium and tritium nuclei lose some of their energy, reducing the number of conversion products which reach the sensor and deposit enough energy to be detected. This will of course not be an issue in the vacuum of space.

The second aspect is the 0.1-micron-thick layer of aluminum on top of the Timepix3 sensor, which serves as a conductor for distribution of the BIAS voltage across the sensor. It has the same effect as the air gap above, reducing the number of detected conversion products. Both issues can, in principle, be eliminated by applying the LiF converter layer directly on the sensor. LiF, being conductive, can perform as the BIAS conductor in place of Al, this option will be further investigated.

## 5. Acquisition Time Estimation

**Figure 15:**
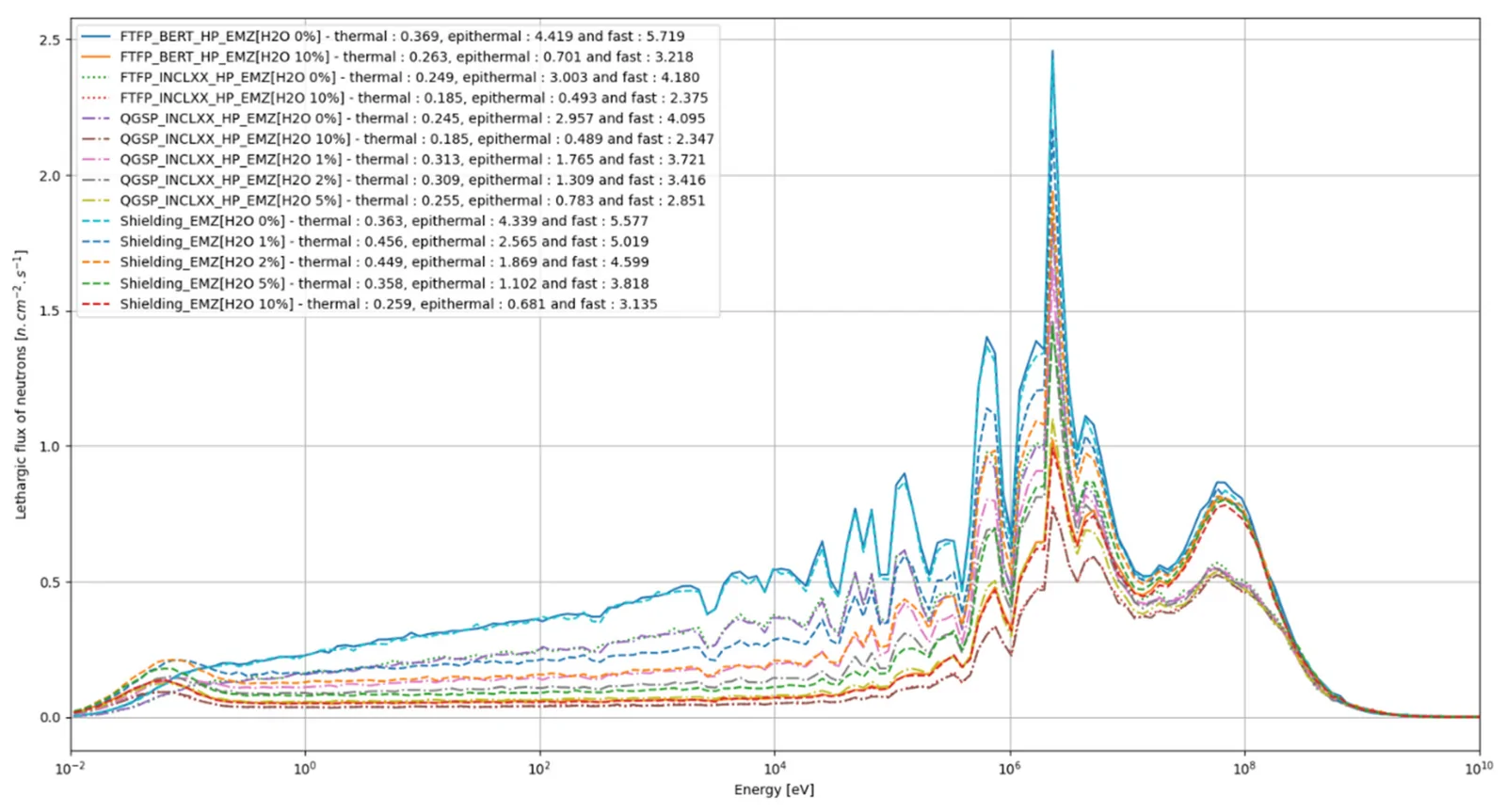
Lethargy fluxes of neutrons based on physic list and water composition, extracted from Figure 3.

Lunar neutron fluxes simulated by DLR with Geant4 were used as input spectra. To improve our understanding of the lunar environment, we ordered the neutron fluence rates based on the physics lists and water percentages. Figure 15 shows the lethargy flux of neutrons from thermal to fast neutrons for different physics lists and water percentages. We observe that the shape of the neutron spectra is similar for all physics lists used during the computation.

Considering that the shapes are similar for all physics lists (and, thus, so are the ratios between thermal, epithermal and fast neutrons) we tried to define the physics lists for the best- and the worst-case scenarios, representing the highest and lowest fluence rates respectively.

**Figure 16:**
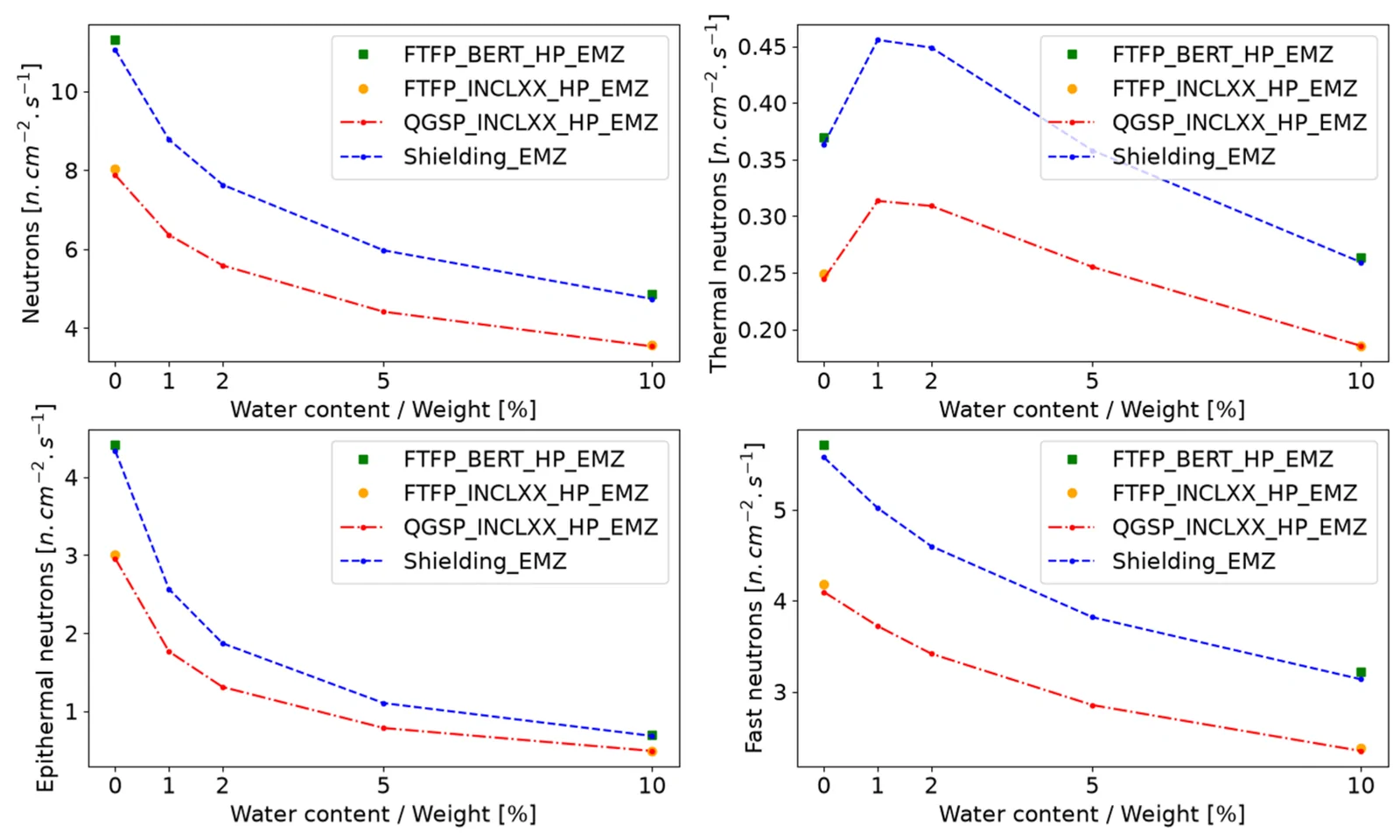
Comparison of the total fluence rate of neutrons (**upper left**), fluence rate of thermal neutrons (**upper right**), epithermal neutrons (**lower left**) and fast neutrons (**lower right**) according to the water content and the physics list used.

Next, we integrated the spectra of neutrons in four different ways: the entire spectra, the thermal neutron spectra, the epithermal neutron spectra and the fast neutron spectra, to compute, respectively, fluence rates of neutrons in total and in each energy band. Figure 16 shows the fluence rates of all thermal, epithermal and fast neutrons based on the water content and on the physics list used.

We observed that the FTFP_BERT_HP_EMZ physics list provided the highest fluxes, but only slightly exceeded the results of the “Shielding” physics list, which is the recommended physics list for neutron transport. We use Shielding_EMZ as the best-case scenario, since it has the second-highest fluence rate. The QGSP_INCLXX_HP_EMZ physics list gives the lowest fluence rates, so it will represent the worst-case scenario.

It is important to note that fluence rates of the best-case scenario are at maximum 50% higher than those of the worst case, and physics list selection does not change fluence rate ratios between different energy bands, meaning that the fluence rates of each physics list translate into acquisition times in a predictable way–higher fluence rate equals proportionally shorter acquisition times.

For the following calculations, we will be using the best-case scenario of the Shielding_EMZ physics list.

### 5.1. Instrument Sensitivity Area and Detection Efficiency

**Figure 17:**
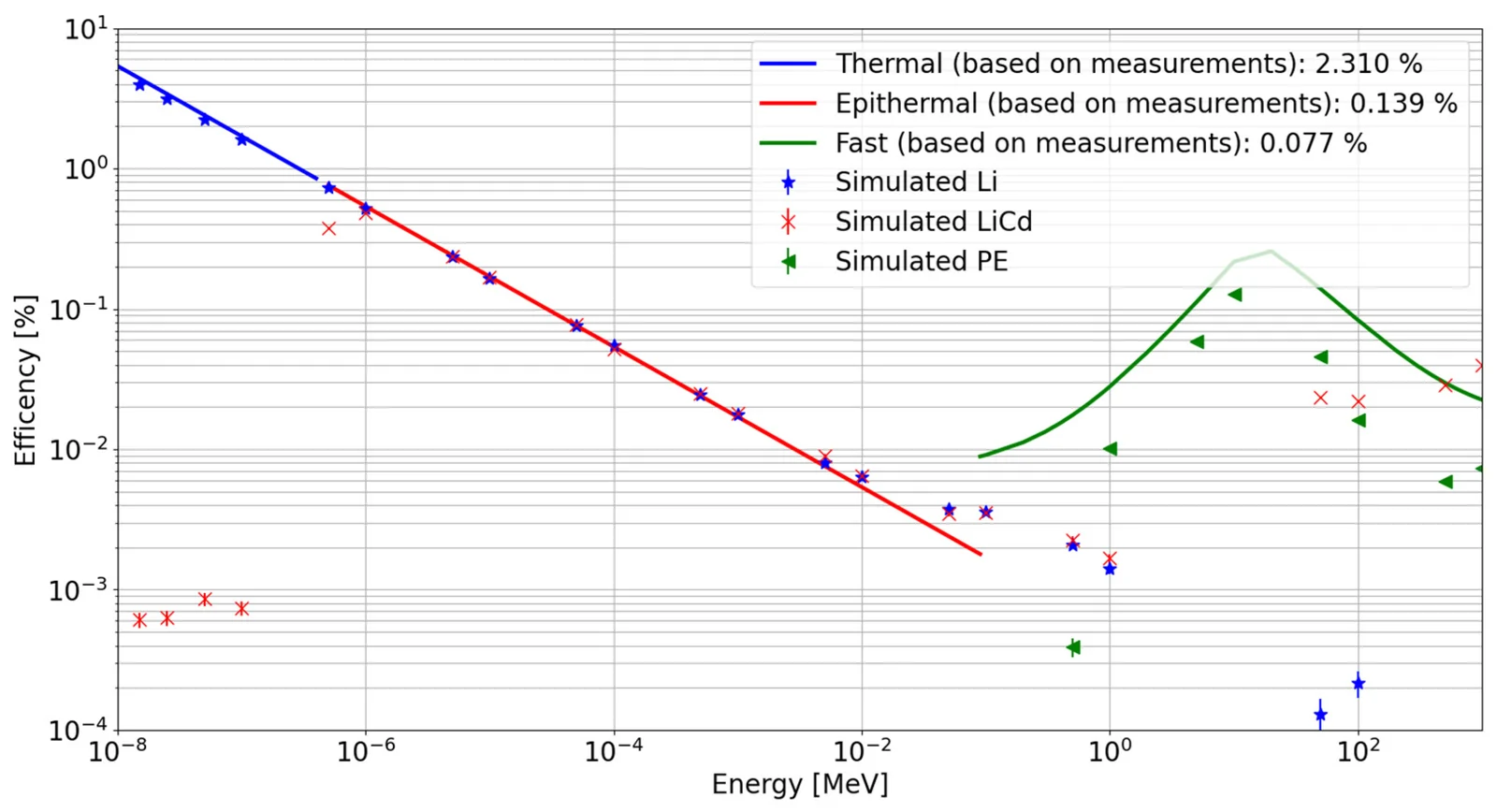
Neutron-detection efficiencies measured (full lines) versus simulated conversion layers (markers) in percentage of water mass ratio in regolith according to neutron energy: blue for thermal, red for epithermal and green for fast neutrons. The small shift in fast-neutron simulation vs. measurement might be explained by the fact that simulations used 2 mm of PE converter while the measurements were conducted using 1 mm of PE.

Two units of Neutron HardPix detectors with two Timepix3 sensors each were used as inputs for detector surface area. One Timepix3 sensor (2 cm^2^) was used for detection of thermal neutrons, one for epithermal neutrons, one for fast neutrons, and one as a reference for the charged particles environment and to subtract the background. Figure 17 shows the neutron-detection efficiencies according to the energy. Efficiencies measured in the previous section are shown as full curves, while efficiencies simulated using detector response functions are shown as markers with the associated Monte Carlo uncertainties.

The simulated LiF layer is sensitive to thermal and epithermal neutrons, with an efficiency very similar to the measured efficiency. The LiF layer with Cd shielding is sensitive to epithermal neutrons but blocks thermal ones. It also shows a small reaction to fast neutrons at very high energies (>50 MeV). Finally, the simulated PE conversion layer is sensitive to fast neutrons, with a slightly lower efficiency than the measured one.

Flux of fast neutrons does not change as radically with hydrogen abundance as the flux of thermal and epithermal neutrons, but we use it to overcome the behavior of thermal neutron flux which increases at first with rising hydrogen abundance and then decreases with higher abundance, making it difficult to distinguish between 0 and 3–5% water ratio using just thermal neutron flux alone. Based on the flux of cosmic ray albedo neutrons calculated for the lunar surface, we estimated the number of neutrons detected by two Neutron HardPix units’ sensors. Based on the counts and ratio of thermal, epithermal and fast neutrons measured in a given time, we can estimate the relative change in the water ratio in the local lunar regolith.

### 5.2. Estimated Count Rates

**Figure 18:**
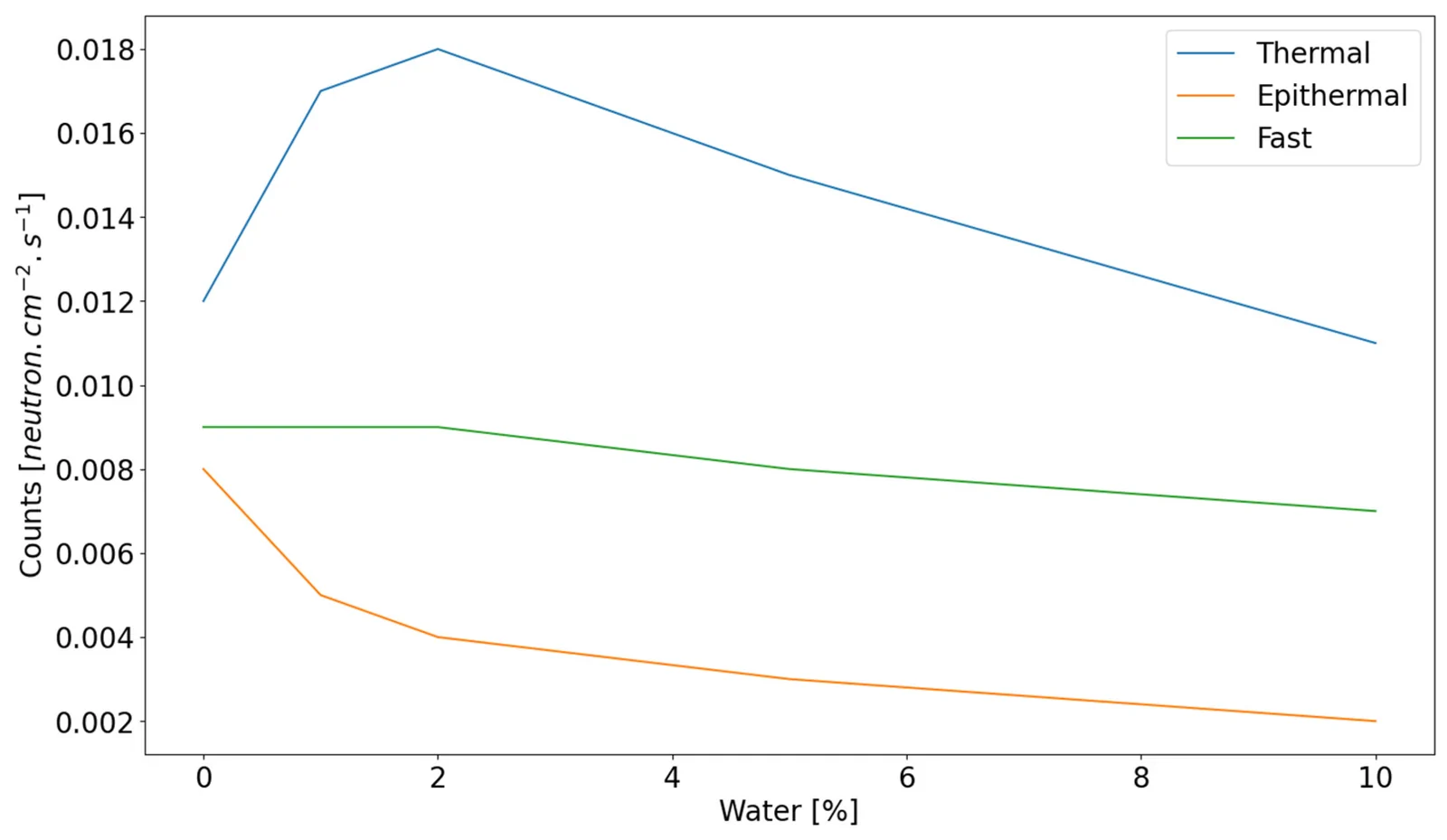
Estimated count rates detected by the Neutron HardPix based on water content and the measured efficiency.

Considering the spectra of neutrons shown in Figure 15 and the measured efficiency shown in Figure 17, we estimated the count rates per unit of detector surface area and time. Figure 18 shows the counts per second per cm^2^ according to the water mass percentage in the regolith.

### 5.3. Estimated Acquisition Times

**Figure 19:**
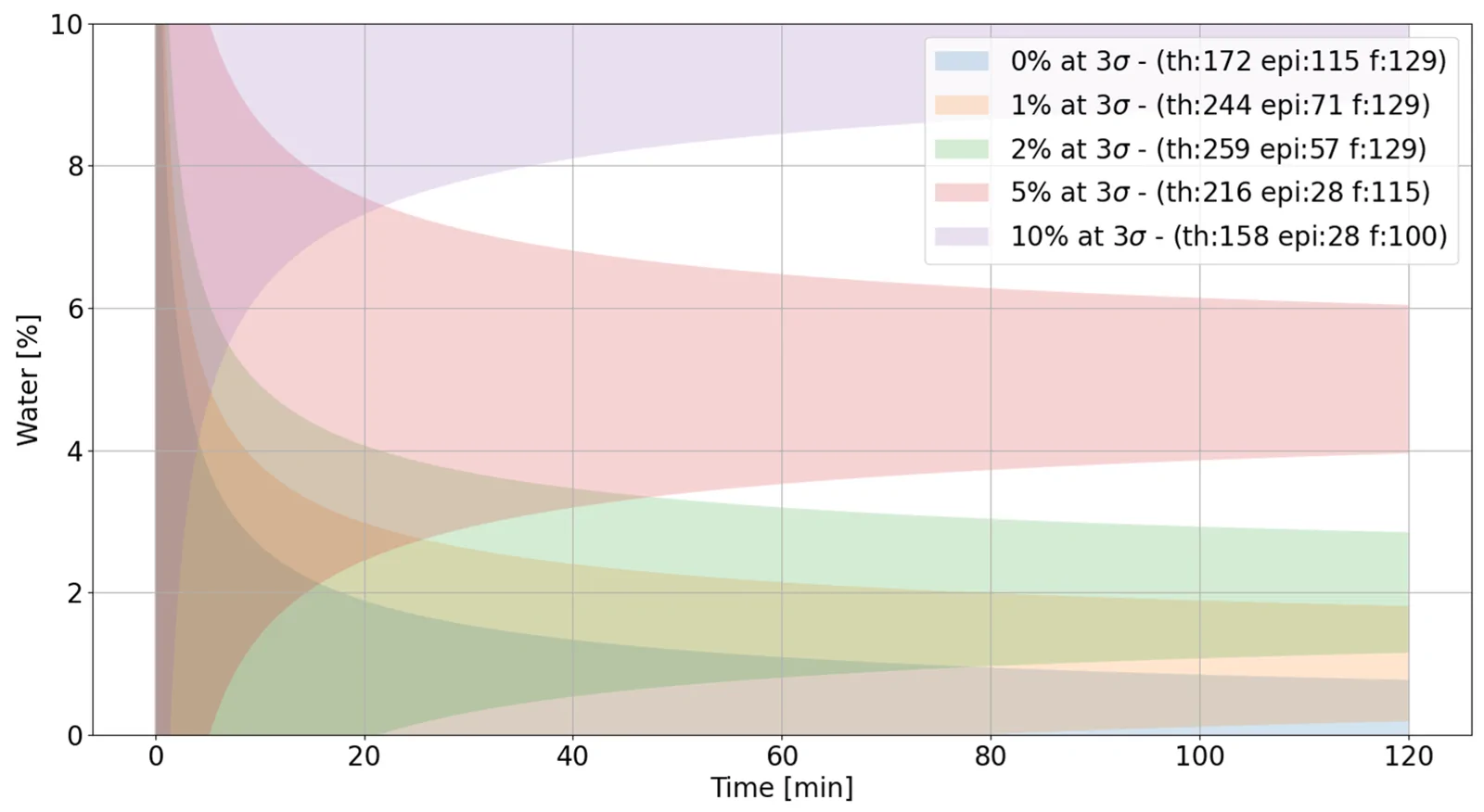
Water content predictions at 3σ precisions according to the number of detected neutrons over measurement time.

Based on the count rates estimated in Figure 18, we estimated the time needed to predict water content using $\sigma =\frac{1}{\sqrt{N_{thermal}}}+\frac{1}{\sqrt{N_{epithermal}}}+\frac{1}{\sqrt{N_{fast}}}$ with a surface area of 2 cm^2^ for each energy bin as described above. This estimation is presented in Figure 19 for 0, 1, 2, 5 and 10% water mass ratio at 3σ. This shows that it should be possible to differentiate 5% water mass ratio change, i.e., between the 0% and 5%, and between 5% and 10%, in roughly 20 min. Detecting a 3% difference between 2% and 5% water mass ratio takes around 50 min, while a detecting 2% difference between 0% and 2% takes roughly 80 min. Detecting a small difference of 1% between 0% and 1%, or 1% and 2% then takes in the order of several (>2) hours, nicely representing the increasing acquisition times needed to detect smaller changes in lunar regolith water mass ratio. However, these calculations were made considering only the number of neutrons detected, not their weight in the prediction nor the ratio between them.

## 6. Neutron HardPix Hydrogen Detection Measurements

### 6.1. Phantom Materials and Setup

Possible materials for lunar regolith simulants available at the VdG facility included pure silica sand SiO_2_ in 25 L barrels and PE sheets. The goal of these simulants was to recreate a neutron radiation environment on the lunar surface above regolith with different water mass ratios as closely as possible. The best setups were designed based on simulations of the effects of simulants on VdG monoenergetic neutron fields. During these simulations, the limitations of the VdG possibilities became apparent.

- In order to simulate thermal and epithermal neutron fields within the VdG chamber, we had to use the lowest practical beam settings; i.e., 1 MeV neutrons. Moderating enough fast neutrons at higher settings to simulate thermal and epithermal spectra shapes representative of the lunar surface would require large amounts of material, mainly sand, which would be difficult within the timeframe of this project.
- To properly model the fast-neutron part of the lunar spectra, we would need neutrons of energies of at least 10 MeV, ideally up to 1 GeV, though from simulations it seems that neutrons from 10 MeV to 1 GeV are not influenced by hydrogen, so 10 MeV might be enough. For this reason, fast-neutron count rates were not used in the analysis of VdG measurements with the Neutron HardPix breadboard (BB), even though we did measure them.
- Simulations showed that spectra representative of very low water mass ratios (~0–1%) are very difficult to simulate with the setup available at the VdG, and thus these were not tested. This is unfortunate, as the difference in count rates between the dry regolith and small water mass ratios promised to be much larger than between different water mass ratios, and it would have been very interesting to experimentally validate this.

### 6.2. Experimental Setup

**Figure 20:**
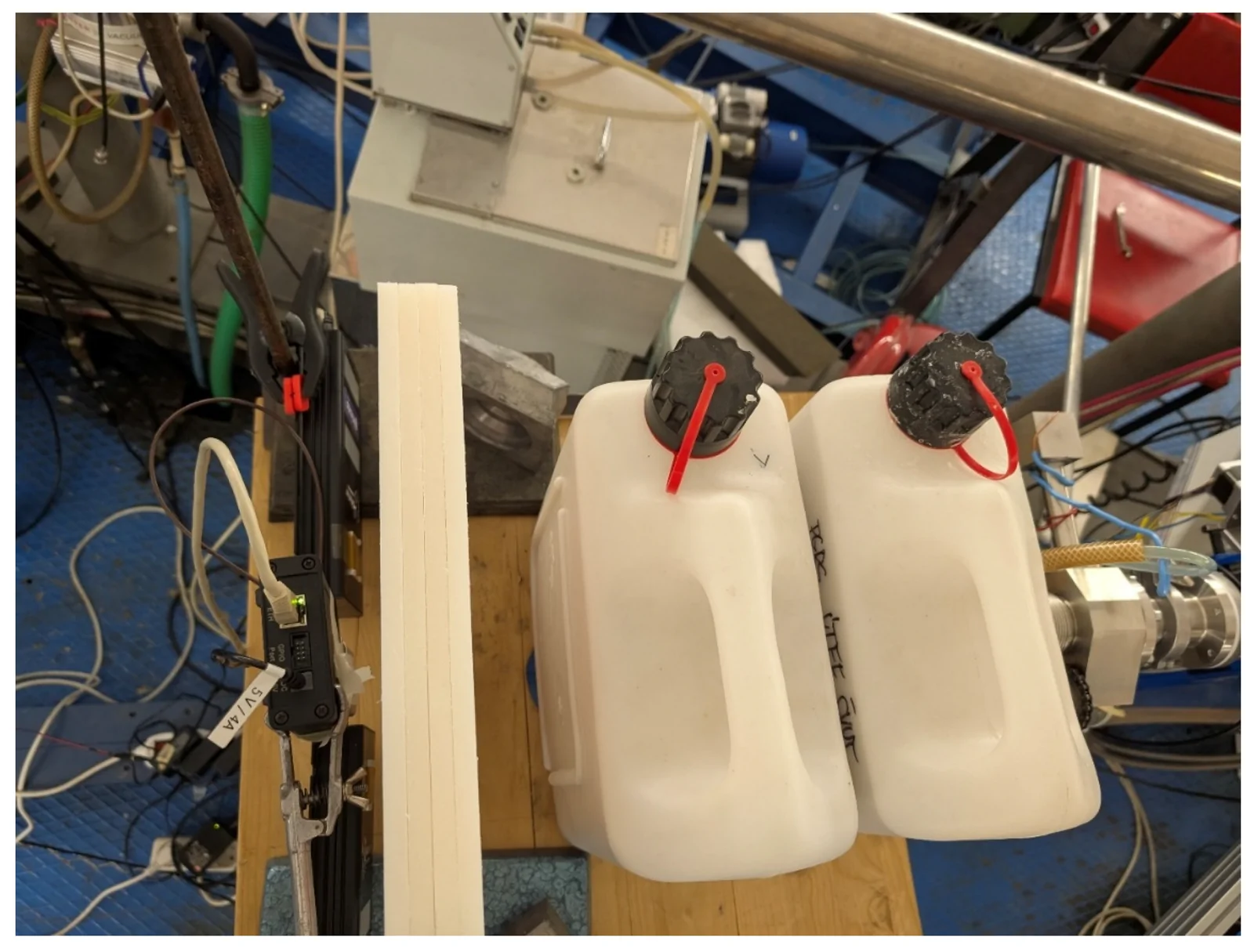
1 MeV neutron beam moderated by 30 cm of SiO_2_ and 1 cm-thick sheets of PE. Detectors with Timepix3 sensor are visible on the left.

Two high-density polyethylene (HDPE) barrels with dimensions of 28 × 28 × 15 cm^3^ and filled with SiO_2_ with density of 1.5 g/cm^3^ were placed in the neutron beam and followed by PE sheets with dimensions of 32 × 32 × 1 cm^3^ and density of 0.960 g/cm^3^ (Figure 20). Measurements were performed with total PE thicknesses of 2, 3, 4 and 5 cm, where HDPE barrels add an extra 2 mm to each wall thickness, i.e., 8 mm of PE total.

Detectors were placed behind these simulants. A Neutron HardPix BreadBoard (BB) with an LiF converter layer was used for thermal + epithermal neutron detection. The same detector was later covered with 1 mm Cd foil to block thermal neutrons and measure epithermal neutron count rate. Two detectors with PE converter from the Measurement and Instrumentation for Cleaning and Decommissioning Operations (MICADO, www.micado-project.eu) project were used to detect fast neutrons, though as stated above, these measurements were not used in the end as the count rate of fast neutrons in the VdG’s lowest settings was not high enough for a meaningful analysis.

**Figure 21:**
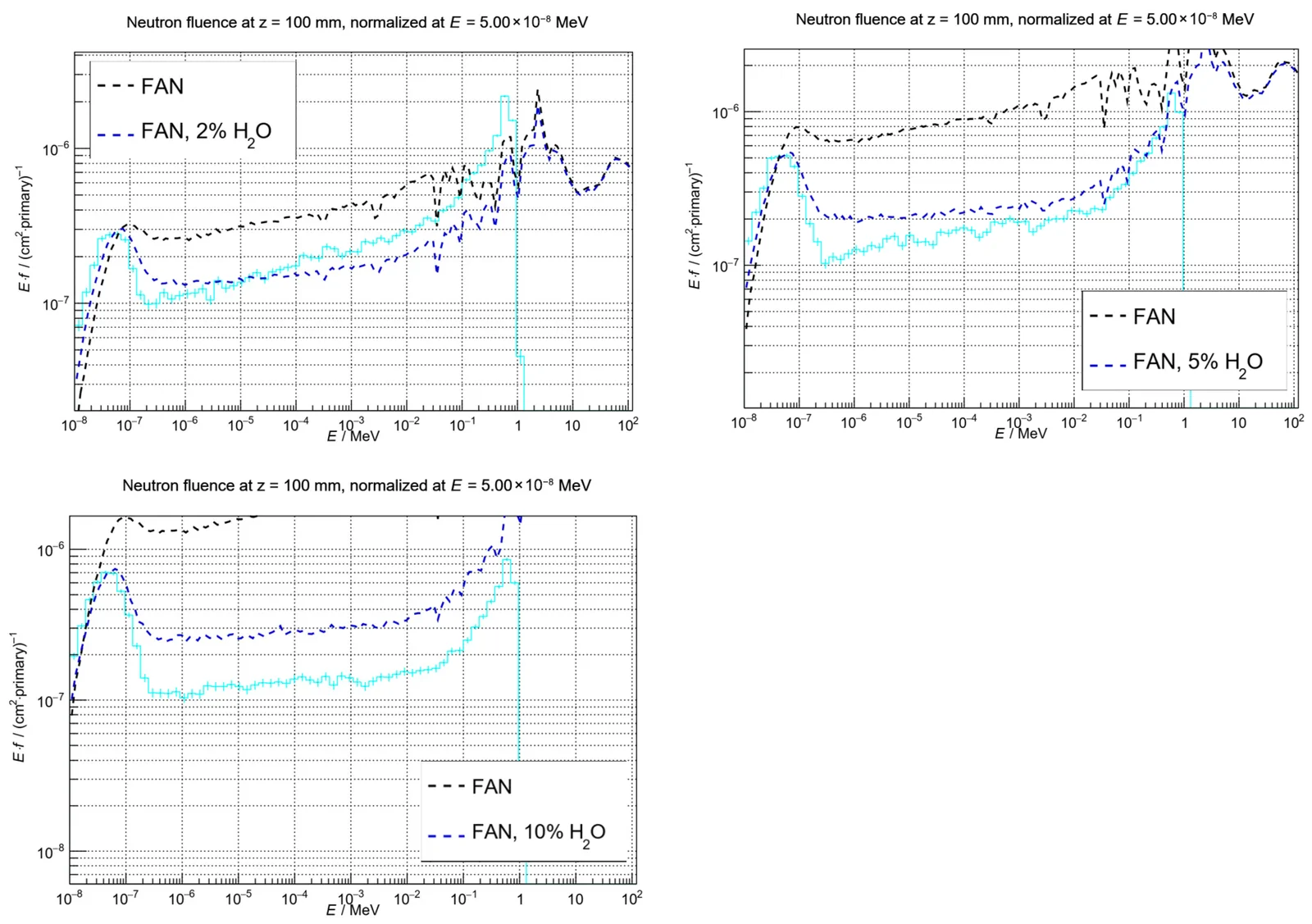
Neutron spectra calculated for the VdG accelerator setup (cyan) compared to the results of the model for the lunar surface, with dry FAN regolith (black) and H_2_O contents in legends (blue). VdG spectra were calculated for simulants with 30 cm SiO_2_ + 2.8 cm PE (**upper left**), 30 cm SiO_2_ + 3.8 cm PE (**upper right**) and 30 cm SiO_2_ + 4.8 cm PE (**lower left**).

**Figure 22:**
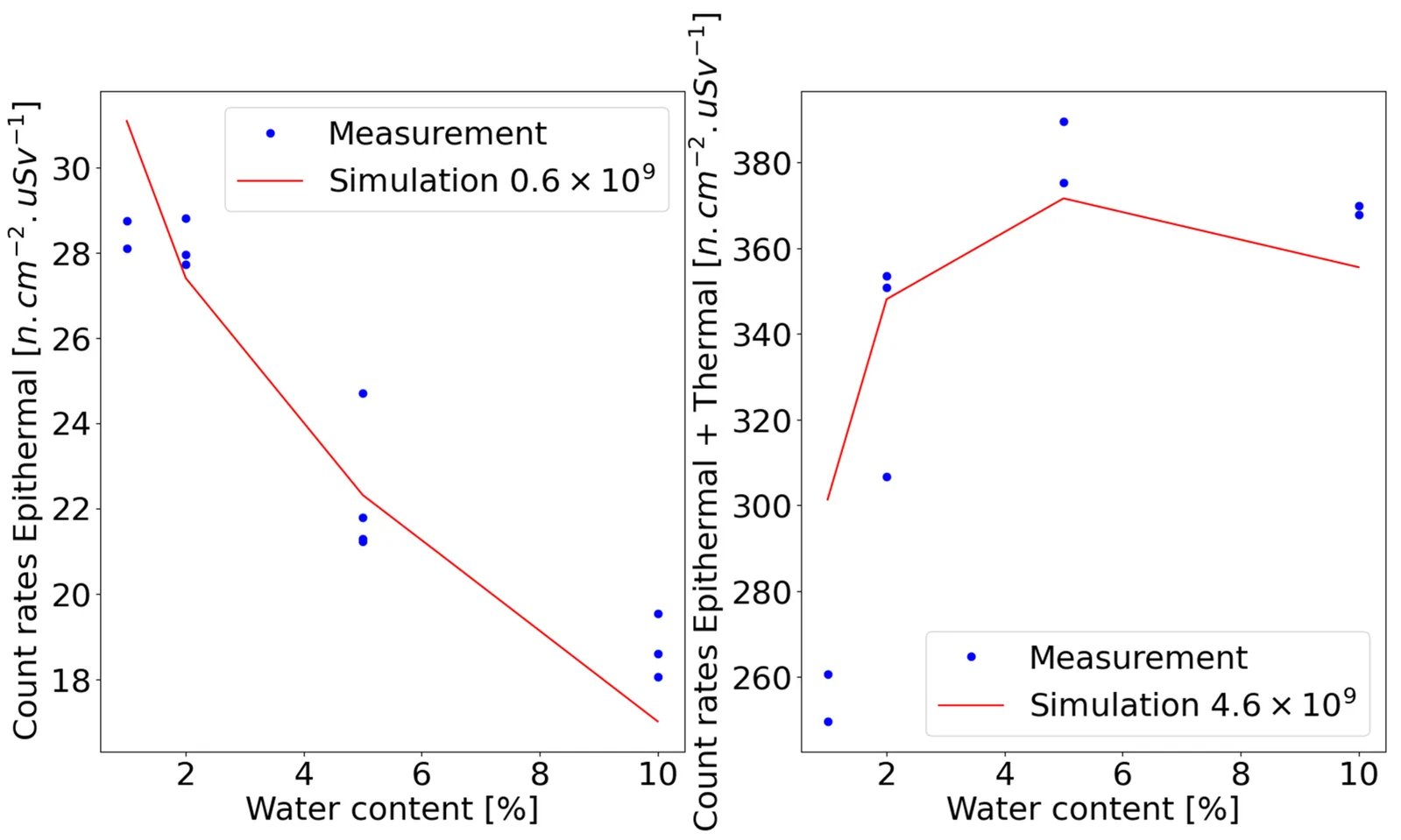
Measured count rates of epithermal (**left**) and thermal + epithermal (**right**) neutrons, shown as blue dots vs. simulated count rates for each setup—30 cm SiO_2_ + 1.8, 2.8, 3.8 and 4.8 cm PE, represented by the regolith with 1, 2, 5 and 10% of water content.

The following experimental setups were modeled and measured by the VdG neutron beam, all using 1 MeV neutrons as a source. The neutron spectra calculated for the lunar surface were normalized to the expected neutron flux at the accelerator at 0.05 eV to facilitate comparison of the shape of the spectra (Figure 21):

The measurements were conducted with 1 h cycles and then normalized with respect to the dose measured by Berthold LB6411 neutron counter/dosemeter. The results of the measurements are summarized in Figure 22, showing measured count rates representing water mass ratios compared to simulations of VdG spectra. These measurements are in good agreement with the simulations; some differences could be explained by the thermal background from walls and surrounding material increasing with larger amounts of PE.

While we were unable to precisely reproduce neutron spectra on top of the lunar regolith at our VdG facility, the neutron environments produced were sufficiently similar to verify the performance of tested neutron spectrometers in thermal and epithermal neutron regions. The differences were caused mainly by the insufficient quantity of material present to sufficiently thermalize fast neutrons, forcing us to use the lowest setting for the VdG, but also backscattering as well as idealistic modeling of the VdG source. Despite these approximations, Neutron HardPix breadboard measurements showed very good agreement with simulations, validating its performance as a neutron spectrometer in the environment expected on the lunar surface. The next steps will include repeating such verification with mechanically representative prototypes of a multilayer Neutron HardPix to validate the setup described above.

## 7. Conclusions

Secondary cosmic-ray-induced neutron spectra on the lunar surface were calculated using Geant4, and the dependence of thermal, epithermal, and fast neutron fluence rates on regolith water content was investigated for different regolith compositions, solar cycle conditions, and transport physics models. These simulations provided the expected neutron environment for the Neutron HardPix instrument and were used as the basis for detector optimization, performance assessment, acquisition time estimates, and the design of laboratory validation measurements. The results indicate the strong sensitivity of the epithermal neutron flux to hydrogen abundance and confirm the suitability of neutron spectroscopy for lunar water prospecting.

The proposed Neutron HardPix instrument combines the space heritage of the HardPix radiation monitor with extensive previous experience in Timepix-based neutron detection. While the underlying neutron detection principles and converter technologies are well established, the novelty of this work lies in the application of a compact semiconductor detector to planetary hydrogen and water mapping, a field that has traditionally relied on ^3^He-based neutron spectrometers. The results suggest that Timepix3-based detectors can provide a viable solid-state alternative for future planetary missions where low mass, compactness, and system simplicity are important considerations.

Experimental measurements performed at calibrated neutron facilities confirmed the expected performance of LiF and polyethylene conversion layers and enabled optimization of the detector configuration. The VdG measurements were used for relative comparison and validation of detector concepts, while absolute thermal neutron efficiency was obtained in a calibrated neutron field at the Czech Metrology Institute. The measured detector response was subsequently combined with simulated lunar neutron spectra to estimate the expected count rates and hydrogen mapping performance.

The acquisition times and water-content sensitivities presented in this work are therefore performance projections derived from lunar environment simulations combined with experimentally measured detector efficiencies. They should not be interpreted as experimentally demonstrated lunar performance. Under the model assumptions used, the results indicate that water-content variations of a few percent could be detectable on timescales of approximately one hour, depending on local conditions and hydrogen abundance.

The present study focuses on validating the measurement concept and the fundamental detector elements. Future work will focus on the development and validation of a flight-representative Neutron HardPix instrument. This includes optimization of the final detector architecture, detailed modeling of the rover structure and surrounding materials, and comprehensive simulations of the mixed radiation environment expected on the lunar surface. Experimental campaigns will be conducted to quantify background contributions from charged particles and secondary radiation, validate the background-subtraction procedures, and establish a complete systematic uncertainty budget.

Neutron HardPix is currently being developed for the ESA-funded commercial lunar rover Mission for Advanced Geophysics and Polar Ice Exploration (MAGPIE) led by ispace Europe, and has recently been approved for implementation. The mission will provide an opportunity to demonstrate semiconductor-based neutron spectroscopy for lunar hydrogen and water mapping under real operational conditions.

## Figures and Tables

**Table 1:** Elemental mass fractions used in the model calculations for the different water contents. The first line represents the regolith without water and was calculated from Table 6.3 (Mare) in [34].

	Elemental Mass Fraction in Regolith								
H_2_O Mass Fraction	H	O	Na	Mg	Al	Si	Ca	Ti	Fe
0.00	0.0000	0.4317	0.0041	0.0555	0.0785	0.2124	0.0843	0.0236	0.1100
0.01	0.0011	0.4363	0.0041	0.0549	0.0777	0.2103	0.0834	0.0233	0.1089
0.02	0.0022	0.4408	0.0040	0.0544	0.0769	0.2082	0.0826	0.0231	0.1078
0.05	0.0056	0.4545	0.0039	0.0527	0.0745	0.2018	0.0801	0.0224	0.1045
0.10	0.0112	0.4773	0.0037	0.0499	0.0706	0.1912	0.0759	0.0212	0.0990

**Table 2:** Neutron fluence rates on the lunar surface from primary GCR calculated for solar minimum conditions and different water contents in the regolith and Geant4 physics list Shielding_EMZ.

	Solar Minimum					
	*E* < 0.48 eV		0.48 eV ≤ *E* < 0.1 MeV		Total	
H_2_O Mass Fraction	*f*/(cm^2^∙s)^−1^	Δ wrt. 0% H_2_O/%	*f*/(cm^2^∙s)^−1^	Δ wrt. 0% H_2_O/%	*f*/(cm^2^∙s)^−1^	Δ wrt. 0% H_2_O/%
0.00	0.409	0.0	4.410	0.0	10.977	0.0
0.01	0.490	19.9	2.615	−40.7	8.719	−20.6
0.02	0.476	16.5	1.907	−56.8	7.582	−30.9
0.05	0.376	−8.0	1.125	−74.5	5.931	−46.0
0.10	0.270	−33.9	0.697	−84.2	4.709	−57.1

**Table 3:** Neutron fluence rates on the lunar surface from primary GCR calculated for solar maximum conditions and different water contents in the regolith and Geant4 physics list Shielding_EMZ.

	Solar Maximum					
	*E* < 0.48 eV		0.48 eV ≤ *E* < 0.1 MeV		Total	
H_2_O Mass Fraction	*f*/(cm^2^∙s)^−1^	Δ wrt. 0% H_2_O/%	*f*/(cm^2^∙s)^−1^	Δ wrt. 0% H_2_O/%	*f*/(cm^2^∙s)^−1^	Δ wrt. 0% H_2_O/%
0.00	0.197	0.0	2.022	0.0	4.782	0.0
0.05	0.164	−16.9	0.472	−76.7	2.459	−48.6
0.10	0.112	−43.2	0.280	−86.1	1.912	−60.0

**Table 4:** Neutron fluence rates on the lunar surface from primary GCR calculated for solar minimum conditions and different water contents in the FAN regolith and layered geometry.

	Solar Minimum					
	*E* < 0.48 eV		0.48 eV ≤ *E* < 0.1 MeV		Total	
	FAN regolith: Geant4 physics list: Shielding_EMZ					
H_2_O mass fraction	*f*/(cm^2^∙s)^−1^	Uncertainty	*f*/(cm^2^∙s)^−1^	Uncertainty	*f*/(cm^2^∙s)^−1^	Uncertainty
0.00	0.6902	0.0032	4.3183	0.0082	10.6359	0.0138
0.01	0.6717	0.0028	2.4882	0.0055	8.3086	0.0110
0.02	0.5950	0.0025	1.8234	0.0043	7.2253	0.0095
0.05	0.4207	0.0019	1.0552	0.0030	5.6163	0.0076
0.10	0.2754	0.0014	0.6511	0.0022	4.4625	0.0065
	Layered FAN regolith: Geant4 physics list: Shielding_EMZ					
H_2_O mass fraction	*f*/(cm^2^∙s)^−1^	uncertainty	*f*/(cm^2^∙s)^−1^	uncertainty	*f*/(cm^2^∙s)^−1^	uncertainty
0.05	0.2854	0.0016	1.4364	0.0037	6.4639	0.0085
0.10	0.2078	0.0013	1.0633	0.0029	5.8167	0.0075

## Data Availability

Data supporting reported results can be found at Zenodo. https://doi.org/10.5281/zenodo.21488210.

## Abbreviations

The following abbreviations are used in this manuscript:

ASIC	Application specific integrated circuit
ATLAS	Toroidal LHC ApparatuS
BB	BreadBoard
BERT	Bertini Intranuclear Cascade
CERN	European Organization for Nuclear Research
CMI	Czech Metrology Institute
COTS	Commercial of the shelf
DAN	Dynamic Albedo of Neutrons
DLR	German Aerospace Center
EMZ	Electromagnetic Option 4
ESA	European Space Agency
FAN	Ferroan ANorthosite
FREND	Fine Resolution Epithermal Neutron Detector
FTFP	Fritiof string + Precompound
GCR	Galactic Cosmic Rays
HDPE	Hogh density polyethylene
HEND	High Energy Neutron Detector
HP	Hogh Precision
IEAP CTU	Institute of Experimental and Applied Physics, Czech Technical University in Prague
IKI	Space Research Institute of the Russian Academy of Sciences
INCL	Liège Intra-Nuclear Cascade
ION	InOrbit Now
ISS	International Space Station
ITAR	International Traffic in Arms Regulations
LANSCE	Los Alamos Neutron Science Center
LEND	Lunar Exploration Neutron Detector
LHC	Large Hadron Collider
LND MAGPIE	Lunar Lander Neutron and Dosimetry Mission for Advanced Geophysics and Polar Ice Exploration
MGNS	Mercury Gamma-ray and Neutron Spectrometer
NASA	National Aeronautics and Space Administration
PE	Polyethylene
PSR	Permanently Shadowed Region
QGSP	Quark-Gluon String + Precompound
SATRAM	Space Application of Timepix-based Radiation Monitor
TGO	Trace Gas Orbiter
ToA	Time of Arrival
VdG	Van de Graaff
WNR	Weapons Neutron Research

## Appendix A

**Table A1:** Absolute values of the neutron fluence rates on the lunar surface from primary GCR calculated for solar minimum conditions and different water contents in the regolith and Geant4 with different physics lists.

	Solar Minimum					
	*E* < 0.48 eV		0.48 eV ≤ *E* < 0.1 MeV		Total	
	*f*/(cm^2^∙s)^−1^	Uncertainty	*f*/(cm^2^∙s)^−1^	Uncertainty	*f*/(cm^2^∙s)^−1^	Uncertainty
H_2_O mass fraction	Geant4 physics list: Shielding_EMZ					
0.00	0.409	0.002	4.410	0.008	10.977	0.014
0.01	0.490	0.002	2.615	0.006	8.719	0.011
0.02	0.476	0.002	1.907	0.004	7.582	0.010
0.05	0.376	0.002	1.125	0.003	5.931	0.008
0.10	0.270	0.001	0.697	0.002	4.709	0.007
H_2_O mass fraction	Geant4 physics list: QGSP_INCLXX_HP_EMZ					
0.00	0.276	0.002	3.007	0.006	7.823	0.010
0.01	0.337	0.002	1.799	0.004	6.319	0.009
0.02	0.328	0.002	1.334	0.003	5.551	0.008
0.05	0.267	0.001	0.799	0.002	4.386	0.006
0.10	0.194	0.001	0.499	0.002	3.514	0.005
H_2_O mass fraction	Geant4 physics list: **FTFP_INCLXX_HP_EMZ**					
0.00	0.281	0.002	3.054	0.008	7.968	0.014
0.10	0.193	0.001	0.504	0.002	3.554	0.007
H_2_O mass fraction	Geant4 physics list: **FTFP_BERT_HP_EMZ**					
0.00	0.414	0.002	4.494	0.008	11.226	0.014
0.10	0.275	0.001	0.716	0.002	4.833	0.006

**Table A2:** Relative differences in the neutron fluence rates on the lunar surface from primary GCR calculated for solar minimum conditions and different water contents in the regolith and Geant4 for different physics lists with respect to Shielding_EMZ.

	Solar Minimum		
	*E* < 0.48 eV	0.48 eV ≤ *E* < 0.1 MeV	Total
	% Difference wrt. Shielding_EMZ	% Difference wrt. Shielding_EMZ	% Difference wrt. Shielding_EMZ
H_2_O mass fraction	Geant4 physics list: **QGSP_INCLXX_HP_EMZ**		
0.00	−32.5	−31.8	−28.7
0.01	−31.2	−31.2	−27.5
0.02	−31.1	−30.0	−26.8
0.05	−28.8	−29.0	−26.1
0.10	−28.3	−28.3	−25.4
H_2_O mass fraction	Geant4 physics list: **FTFP_INCLXX_HP_EMZ**		
0.00	−31.3	−30.7	−27.4
0.10	−28.5	−27.6	−24.5
H_2_O mass fraction	Geant4 physics list: **FTFP_BERT_HP_EMZ**		
0.00	1.5	1.9	2.3
0.10	1.7	2.8	2.6

**Table A3:** Elemental mass fractions used in the model calculations for the different water contents. The first line represents the FAN regolith without water [35,40].

	Elemental Mass Fraction in FAN Regolith								
H_2_O Mass Fraction	H	O	Na	Mg	Al	Si	Ca	Ti	Fe
0.00	0.0000	0.4560	0.0045	0.0051	0.1763	0.2066	0.1359	0.0008	0.0148
0.01	0.0011	0.4603	0.0045	0.0050	0.1745	0.2045	0.1345	0.0008	0.0147
0.02	0.0022	0.4646	0.0044	0.0050	0.1728	0.2025	0.1332	0.0008	0.0145
0.05	0.0056	0.4776	0.0043	0.0048	0.1675	0.1963	0.1291	0.0008	0.0141
0.10	0.0112	0.4992	0.0041	0.0046	0.1587	0.1859	0.1223	0.0007	0.0133
